# Detection of an Intermediate in the Unfolding Process of the N-Terminal Domain of TDP-43

**DOI:** 10.1021/acsomega.4c08617

**Published:** 2025-02-05

**Authors:** Isabella Marzi, Giuseppe Pieraccini, Francesco Bemporad, Fabrizio Chiti

**Affiliations:** † Department of Experimental and Clinical Biomedical Sciences “Mario Serio”, Section of Biochemistry, University of Florence, Viale Morgagni 50, Florence 50134, Italy; ‡ CISM-Mass Spectrometry Centre, University of Florence, Via Ugo Schiff 6, Sesto Fiorentino, Florence 50019, Italy

## Abstract

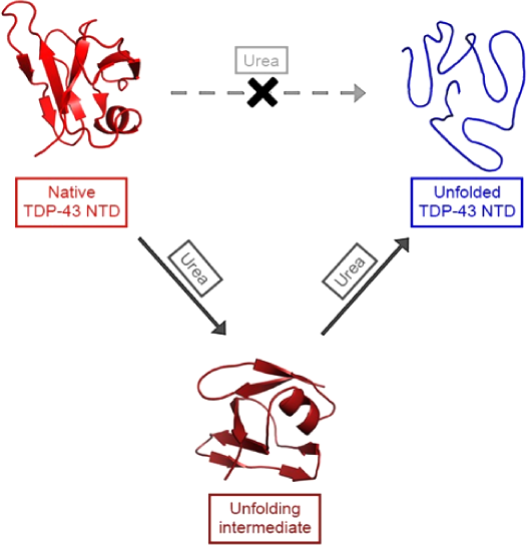

TAR DNA-binding protein 43 (TDP-43) is a nuclear protein accumulating in intraneuronal cytoplasmic inclusions associated with amyotrophic lateral sclerosis, frontotemporal lobar degeneration with tau-negative/ubiquitin-positive inclusions, and limbic-predominant age-related TDP-43 encephalopathy. Oligomerization of full-length TDP-43, driven by its N-terminal domain (NTD), is essential for its function, but aberrant self-assembly also promotes liquid–liquid phase separation and formation of solid inclusions. Building on recent all-atom molecular dynamics simulations and using various biophysical approaches, we identified a partially unfolded state accumulating during unfolding of TDP-43 NTD, before the major energy barrier of unfolding is crossed. Intrinsic fluorescence spectroscopy coupled to a stopped-flow device at high urea concentration reveals that the intermediate state has a fluorescence emission distinct from those of the native and unfolded states and forms within the 14 ms dead time. Conventional fluorescence spectroscopy shows it still accumulates at moderate urea concentration. Circular dichroism and H/D exchange results show a species with an intermediate content of secondary structure and a distorted β-sheet, whereas SYPRO orange fluorescence indicates an open conformation with more exposed hydrophobic regions compared to the native state. Importantly, this intermediate is observed even at low protein concentration, when TDP-43 NTD is largely monomeric, indicating that its formation is independent of the initial TDP-43 NTD oligomeric state. Dynamic light scattering at high protein concentration shows that the intermediate is a partially folded dimer. The intermediate forms upon chemical denaturation and does not occur under thermal unfolding. Overall, the findings highlight the presence of one more partially folded state for TDP-43 NTD, underlining its high structural plasticity and suggesting that its distinct unfolding pathway may play a critical role in both its functional and pathological behaviors.

## 1 Introduction

TAR DNA-binding protein 43 (TDP-43) is a nuclear DNA/RNA binding protein involved in regulation of transcription, pre-mRNA splicing, mRNA stabilization and transport, mRNA translational regulation and other processes associated with RNA metabolism.^1,2^ In 2006, TDP-43 was found to form neuronal cytoplasmic inclusions (NCIs) in the neurons of the central nervous system in neurodegenerative diseases.^3−5^ In some of these disorders, including sporadic and familial forms of amyotrophic lateral sclerosis (ALS), frontotemporal lobar degeneration with tau-negative, ubiquitin-positive inclusions (initially named FTLD-U and now referred to as FTLD-TDP), and limbic-predominant age-related TDP-43 encephalopathy neuropathological change (LATE-NC), the NCIs are thought to play a key role in the etiopathogenesis of the disease, as they are inherently toxic (gain-of-function) and sequester nuclear native TDP-43 with consequent loss of this protein in its native environment (loss-of-function).^6−10^ TDP-43 NCIs are also frequently detected in the neurons of the central nervous system of cases with Parkinson disease,^11^ Huntington disease,^12^ Alzheimer disease,^13^ Creutzfeldt-Jacob disease,^14^ and other neurodegenerative disorders,^15^ suggesting it has a high propensity to aggregate in contexts of failure of the proteostasis network (PN).

From a structural viewpoint, TDP-43 is a complex protein with 414 amino acid residues and includes four distinct domains: a folded N-terminal domain (NTD_1–76_), two RNA recognition motifs (RRM1_106–176_, and RRM2_191–259_), and a largely unstructured C-terminal domain (CTD_274–414_).^16−21^ TDP-43 was initially proposed to be natively dimeric, or at least to exist in a monomer–dimer equilibrium under normal physiological conditions, with dimerization mediated by the NTD.^22−25^ Following the elucidation of the TDP-43 NTD structure at high resolution, with nuclear magnetic resonance (NMR) spectroscopy and X-ray crystallography, it was found that the monomer-to-monomer interface involved distinct structural regions in the first and second monomer and that such an interaction could be propagated to involve other subunits in a head-to-tail fashion up to oligomers with indefinite numbers of units.^26,27^ The dimer dissociation constant*(K*_D_) was found to be 2.4 μM in two independent studies at pH 7.4–7.5,^28,29^ which becomes 40 μM for the oligomer in an isodesmic self-association model at pH 6.8.^27^

The NTD-mediated oligomerization of the full-length protein was found to have a functional role, because mutations destabilizing the NTD-NTD interface without modifying the folded structure of the NTD monomer inhibit the splicing activity of full-length TDP-43 in cells.^26,27^ Although NTD-mediated TDP-43 oligomerization is essential for TDP-43 function, an uncontrolled oligomerization process can also lead to aberrant liquid droplets, through a process of liquid–liquid phase separation (LLPS),^27,30,31^ as well as solid phase inclusions.^24,32^

The NTD monomer consists of a single α-helix and of seven-eight β-strands arranged in an axin-1 DIX domain fold,^21,26,27,33,34^ which facilitates dimer/oligomer formation by a head-to-tail arrangement of individual monomeric subunits.^26,27,35^ The conformational stability of the domain, determined using intrinsic fluorescence and far-UV circular dichroism as optical probes at pH 7.4 and 25 °C in equilibrium urea denaturation curves, was found to be 20.1 ± 1.5 kJ mol^–1^ and 21.8 ± 1.5 kJ mol^–1^, respectively.^35^ The α-helix, β-strands, and three out of four turns appear to be rigid, with only the loop contributed by residues 47–53 appearing mobile.^33^ All X-Pro peptide bonds adopt a *trans* configuration and the two cysteine residues at positions 39 and 50 are reduced and distantly separated on the surface of the protein.^21,26,27,33^ Moreover, the protein domain has a tryptophan residue at position 68, which is a useful intrinsic probe to monitor its conformational change through fluorescence.

The importance of the NTD of TDP-43 in functional dimerization/oligomerization and in pathological LLPS and solid inclusion formation parallels the high structural plasticity of this globular domain. The investigation of the folding process of the NTD from a urea-unfolded state has revealed a number of conformational states sufficiently stable to be populated transiently during folding, including a rapidly formed collapsed state formed on the ms or sub-ms time scale (<6 ms), an on-pathway intermediate state formed on the time scale of ca. 0.1 s and a fully folded monomeric state before the final native dimer/oligomer can be adopted. Addition of small amounts of urea, under conditions in which the protein is a folded dimer, has led to a stable native-like folded dimer in which the native β-sheet twisting is subtly distorted.^35^ Moreover, addition of moderate concentration of Sulfobetaine 3–10 led to the formation of an alternative, cooperatively folded monomeric state enriched with α-helical structure and depleted in β-sheet content.^36^ More generally, the various NMR reports on TDP-43 NTD have shown spectral differences under the various experimental conditions in which the protein domain is folded.^19,21,23,27,33,34^ All these reports lend support to the structural plasticity of the TDP-43 NTD and to its ability to adopt different conformational states.

Along the same lines of the inherent structural mutability of the TDP-43 NTD, all-atom molecular dynamics (MD) simulations of TDP-43 NTD unfolding, carried out previously at high temperature and in the presence of either 8 M urea or 8 M DMSO as denaturing agents, have revealed the formation of relatively stable partially unfolded states differing in their free energy.^37,38^ Accessibility of partially unfolded states from the native structure of the NTD of TDP-43 may play an important role in functional dimerization/oligomerization, but also in pathological LLPS and solid NCI formation, as these conformational states would have a higher propensity to undergo such aberrant and uncontrolled processes.^37,38^ However, formation of partially unfolded states during TDP-43 NTD unfolding awaits experimental confirmation and unfolding intermediates are very rarely observed experimentally in unfolding studies of small globular proteins, where the loss of the folded compact state is generally found to be cooperative and occur kinetically in a monoexponential single-step process. For this reason, we have investigated the unfolding process of the TDP-43 NTD experimentally at both moderate and high urea concentrations, under conditions of neutral pH and room temperature, which means under conditions in which unfolding intermediates are rarely observed.

We will show that the fully folded dimeric domain forms rapidly (<14 ms) a partially unfolded state in urea concentrations in which the native state is destabilized and the protein domain adopts at equilibrium an unfolded state for 100% of its molecules. This intermediate state is detectable with a variety of probes, maintains a dimeric structure and forms before the major unfolding step across the major unfolding free energy barrier. More generally, the results describe a kinetic, partially unfolded dimeric state during the unfolding process, which has not been observed, to our knowledge, in protein unfolding studies, contributing to highlight the structural plasticity of the TDP-43 NTD.

## 2 Materials and Methods

### 2.1 Gene Cloning, Expression, and Purification

Gene cloning, expression, and purification of TDP-43 NTD were performed as previously described.^35^ Purified TDP-43 NTD contained 77 residues with the addition of the MHHHHHHSSGVDLGTENLYFQS sequence at the N-terminus before Met1, for a total of 99 residues. It was stored at 1.6–2.7 mg/mL (150–250 μM) in 5 mM sodium phosphate buffer, 50 mM NaCl, 1 mM DTT, pH 7.4, −20 °C. For a single experiment, the additional sequence at the N-terminus was cleaved as previously described,^35^ yielding a TDP-43 NTD variant containing 78 residues, which includes the last serine residue of the tag. Protein purity was checked with SDS-PAGE. Protein concentration was measured with a SHIMADZU UV-1900 UV–vis spectrophotometer at 280 nm with extinction coefficients (ε_280_) of 11,460 and 12,950 M^–1^ cm^–1^ (assuming all Cys residues are reduced) and molecular weights of 8,615.64 and 11,081.29 Da for cleaved and noncleaved TDP-43 NTD, respectively.

### 2.2 Stopped-Flow Fluorescence Spectroscopy

Intrinsic fluorescence of TDP-43 NTD during the unfolding process was followed in real-time at 25 °C using a Bio-Logic (Claix, France) SFM-3 stopped-flow device, equipped with an FC-08 cuvette, coupled to a fluorescence detection system. The excitation wavelength was 280 nm and a band-pass filter to cut fluorescence emitted below 320 nm was used. The dead time was generally 14.4 ms. The TDP-43 NTD sample was centrifuged at 18,000 *g*, 15 min, 4 °C and incubated at 0.6 mg/mL (54 μM) in 5 mM sodium phosphate buffer, 50 mM NaCl, 1 mM DTT, pH 7.4, 25 °C. Subsequently, to monitor TDP-43 NTD unfolding kinetics, 20 μL of the protein domain sample were mixed in the stopped-flow device with 380 μL of 5 mM sodium phosphate buffer, 50 mM NaCl, 1 mM DTT, 10 M urea, pH 7.4, to reach final conditions of 0.03 mg/mL (2.7 μM) protein, in 5 mM sodium phosphate buffer, 50 mM NaCl, 1 mM DTT, 9.5 M urea, pH 7.4, 25 °C. The traces obtained were blank subtracted. The fluorescence emission (*F*) was plotted as a function of time (*t*) and fitted to an equation that combines a linear and an exponential function:
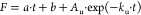
1where *A*_u_ and *k*_u_ are the amplitude and the rate constant of the unfolding process, respectively; *a* and *b* are the coefficients of the straight line.

In another experiment, the TDP-43 NTD sample was centrifuged at 18,000 *g*, 15 min, 4 °C and incubated at 0.6 mg/mL (54 μM) in 5 mM sodium phosphate buffer, 50 mM NaCl, 1 mM DTT, pH 7.4. 20 μL of the sample containing the protein domain were mixed in the stopped-flow device with varying volumes of two solutions of 5 mM sodium phosphate buffer, 50 mM NaCl, 1 mM DTT, pH 7.4, with or without 10 M urea. Final conditions were 0.03 mg/mL (2.7 μM) protein in 5 mM sodium phosphate buffer, 50 mM NaCl, 1 mM DTT, pH 7.4, 25 °C, with urea concentrations ranging from 7.0 to 9.5 M. The resulting traces were blank subtracted and fitted using eq 1. The obtained ln(*k*_u_) values were plotted vs urea concentration, together with the values of TDP-43 NTD treated with 4.5–7.0 M urea obtained in a previous study carried out in our laboratory.^35^ These values were then plotted as a function of urea concentration to create the unfolding arm of the so-called chevron plot. ln(*k*_u_) values from 4.5 to 7.0 M urea and from 7.0 to 9.5 M urea were fitted separately to two different linear equations. Altogether, all data from 4.5 to 9.5 M urea were also fitted to a second order polynomial equation.

### 2.3 Intrinsic Fluorescence Spectroscopy

TDP-43 NTD was centrifuged at 18,000 *g*, 15 min, 4 °C and diluted to 0.5 mg/mL (45 μM) in 5 mM sodium phosphate buffer, 50 mM NaCl, 1 mM DTT, pH 7.4. Fluorescence spectra were acquired using an Agilent Cary Eclipse spectrofluorometer (Agilent Technologies, Santa Clara, CA, USA) equipped with a thermostated cell holder attached to an Agilent PCB 1500 water Peltier system. Data were collected at 25 °C using a 3 × 3 mm black wall quartz cell from 290 to 500 nm (excitation at 280 nm), with excitation and emission slits of 5 nm. Spectra were then blank subtracted.

In another experiment, the TDP-43 NTD sample was centrifuged at 18,000 *g*, 15 min, 4 °C and incubated in the presence of urea at concentrations ranging from 0.0 to 2.5 M, at 0.2 mg/mL (18 μM), in 5 mM sodium phosphate buffer, 50 mM NaCl, 1 mM DTT, pH 7.4, 25 °C. Data were collected at 25 °C using a 10 mm path length quartz cell from 290 to 450 nm (excitation at 280 nm) with excitation and emission slits of 5 nm and using the same Agilent Cary Eclipse spectrofluorometer described above. Spectra were then blank subtracted. Subsequently, the intrinsic fluorescence emission values of TDP-43 NTD in 0.0–2.5 M urea at a given wavelength were plotted as a function of urea concentration and fitted to a linear equation. This was done at all wavelength values. The obtained linear equations were then used to extrapolate the fluorescence emission values of native TDP-43 NTD at 9.5 M urea. The values extrapolated were plotted as a function of wavelength to reconstruct the spectrum of TDP-43 NTD in the native state in 9.5 M urea. The spectrum obtained was then compared with that recorded experimentally when TDP-43 NTD populates the unfolded state in the presence of 9.5 M urea. The area under the spectra for emission wavelength values greater than 320 nm was calculated for both spectra and the value obtained for TDP-43 NTD in the unfolded state was then used as a reference to normalize its unfolding kinetic traces in 9.5 M urea obtained with the stopped-flow device.

Intrinsic fluorescence emission and the Agilent Cary Eclipse spectrofluorometer were also used to monitor TDP-43 NTD kinetics during the unfolding process. TDP-43 NTD was centrifuged at 18,000 *g*, 15 min, 4 °C and incubated in the presence of urea at concentrations ranging from 0.0 to 2.5 M, in 5 mM sodium phosphate buffer, 50 mM NaCl, 1 mM DTT, pH 7.4, 25 °C. In two separate experiments, different final protein concentrations were used, in particular 0.2 mg/mL (18 μM) and 0.0055 mg/mL (0.5 μM). Data were collected at 25 °C using a 10 mm path length quartz cell with excitation and emission wavelengths of 280 and 319 nm, respectively. The excitation and emission slits were 5 nm. The resulting values were blank subtracted. The fluorescence emission (*F*) of TDP-43 NTD in the native state was calculated for each urea concentration, plotted as a function of urea concentration and fitted to a linear equation. The obtained fitting curve was used to extrapolate the fluorescence emission value of TDP-43 NTD of the native state in 4.5 M urea. Subsequently, to measure TDP-43 NTD unfolding kinetics, the protein domain at a final protein concentration of 0.2 mg/mL (18 μM) or 0.0055 mg/mL (0.5 μM) was incubated with 4.5 M urea in 5 mM sodium phosphate buffer, 50 mM NaCl, 1 mM DTT, pH 7.4, 25 °C. The dead time was generally 10–15 s. The resulting traces were blank subtracted and fitted to a single exponential equation of the type:
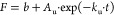
2where *b*, *A*_u_, and *k*_u_ have the same meaning as in eq 1.

### 2.4 SYPRO Orange Fluorescence

SYPRO Orange dye 5000X (Thermo Fisher Scientific) was diluted 20-fold in DMSO to get a 250X solution. The unfolding process of TDP-43 NTD was monitored over time in 400 μL under the same condition described above (5 mM sodium phosphate buffer, 50 mM NaCl, 1 mM DTT, pH 7.4, 25 °C) with a final protein concentration of 0.2 mg/mL (18 μM), with the addition of 1.6 μL SYPRO Orange dye 250X down to a final dye concentration of 1X. Data were collected over time at 25 °C using a 10 mm path length quartz cell with excitation and emission wavelengths of 472 and 595 nm, respectively, using the same Agilent Cary Eclipse spectrofluorometer described above. The excitation and emission slits were 10 nm. The dead time was generally 10–15 s. The resulting traces were blank subtracted. The fluorescence emission (*F*) of SYPRO Orange when TDP-43 NTD populates the native state was calculated for urea concentrations ranging from 0.0 to 2.5 M. It was then plotted as a function of urea concentration and fitted linearly to extrapolate the fluorescence emission value of SYPRO Orange when TDP-43 NTD is in the native state in the presence of 4.5 M urea. To measure TDP-43 NTD unfolding kinetics using SYPRO Orange as a probe, the protein domain was incubated at a final protein concentration of 0.2 mg/mL (18 μM) with 4.5 M urea and SYPRO Orange 1X under the same conditions as above. The resulting traces were blank subtracted and fitted to eq 1.

### 2.5 Far-UV Circular Dichroism (Far-UV CD) Spectroscopy

TDP-43 NTD was centrifuged at 18,000 *g*, 15 min, 4 °C and diluted to 0.5 mg/mL (45 μM) in 5 mM sodium phosphate buffer, 50 mM NaCl, 1 mM DTT, pH 7.4. Spectra were acquired using a Jasco J-810 spectropolarimeter (Tokyo, Japan) equipped with a thermostated cell holder attached to a Julabo CORIO CD 200F water bath (Seelbach, Germany). Data were collected between 190 and 260 nm at 25 °C using a 0.1 mm path length cell. Spectra were then blank subtracted, truncated when the high tension (HT) was higher than 700 V and normalized to mean residue ellipticity using
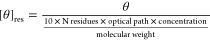
3where [θ]_res_ is the mean residue molar ellipticity in deg cm^2^ dmol^–1^, θ is the ellipticity in mdeg, optical path is in cm, concentration is in g/L, and molecular weight is in g/mol.

Far-UV CD was also used to monitor in real time TDP-43 NTD unfolding. TDP-43 NTD was centrifuged at 18,000 *g*, 15 min, 4 °C and incubated with low urea concentrations, to final values ranging from 0.0 to 2.5 M, at 0.2 mg/mL (18 μM) in 5 mM sodium phosphate buffer, 50 mM NaCl, 1 mM DTT, pH 7.4, 25 °C. Data were collected at 25 °C from 226 to 240 nm, using a 1 mm path length cell, blank subtracted and normalized to [θ]_res_ using eq 3. [θ]_res_ was then plotted as a function of urea concentration and fitted to a linear equation to extrapolate the [θ]_res_ value of the native state in 4.5 M urea. To measure TDP-43 NTD unfolding kinetics, the protein domain at a final protein concentration of 0.2 mg/mL (18 μM) was incubated with 4.5 M urea under the same conditions. The traces obtained were blank subtracted and normalized using eq 3. [θ]_res_ was plotted against time and fitted to a single exponential equation of the type:
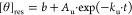
4where *b*, *A*_u_, and *k*_u_ have the same meaning as in eqs 1 and 2.

### 2.6 Hydrogen–Deuterium Exchange Mass Spectrometry (HDX-MS)

TDP-43 NTD was centrifuged at 18,000 *g* for 15 min at 4 °C and the supernatant was diluted in a 1:5 (v/v) ratio with liquid chromatograph for mass spectrometry (LC-MS) grade water. For native conditions, TDP-43 NTD was mixed at 0 °C with D_2_O in a 1:1 (v/v) ratio and incubated for 15 s before quenching by adding 1% trifluoroacetic acid (TFA), resulting in a final pH of ∼2. For denaturing conditions, the protein was diluted 1:1 (v/v) at 0 °C with D_2_O and 9 M urea-*d*_4_ and quenched after 15 s or after 3 min of incubation by adding 1% TFA. The exchanged TDP-43 NTD solutions were prepared for mass spectrometry by adding acetonitrile and infused into the ESI Ion Max interface of a calibrated LTQ Orbitrap mass spectrometer (Thermo Fisher Scientific, Waltham, MA, USA). Mass spectra were acquired by summing the MS scans over the range of 800–2000 *m*/*z* for 0.15 min, operating at a resolution of 100,000 (at 400 *m*/*z*). Data acquisition and analysis were performed using Xcalibur software (Thermo Fisher Scientific, Waltham, MA, USA).

### 2.7 Dynamic Light Scattering (DLS)

The TDP-43 NTD sample was centrifuged at 18,000 *g*, 15 min, 4 °C, filtered with Whatman Anotop filters having a cutoff of 20 nm (Merck) and diluted to 0.5 mg/mL (45 μM) in 5 mM sodium phosphate buffer, 50 mM NaCl, 1 mM DTT, pH 7.4. Its size distribution (distribution of apparent hydrodynamic diameter by light scattering intensity) was acquired using a Malvern Panalytical Zetasizer Nano S DLS device (Malvern, Worcestershire, UK), thermostated at 25 °C with a Peltier temperature controller. Data were collected at 25 °C using a 3 × 3 mm black wall quartz cell with the cell position 4.20 and attenuator index 10. A 2WAJ ABBE bench refractometer from Optika Microscopes (Bergamo, Italy) and a Viscoball viscometer (Fungilab, Barcelona, Spain) were used to acquire the refractive index and viscosity, which were 1.331 and 0.8998 cP, respectively.

DLS was also used to monitor TDP-43 NTD size distribution during the unfolding process. The TDP-43 NTD sample was centrifuged at 18,000 *g*, 15 min, 4 °C, filtered with Whatman Anotop 0.02 μm cutoff filters and incubated with urea to a final concentration of 4.5 M. The final protein concentration was 0.5 mg/mL (45 μM). The hydrodynamic diameter (*D*_H_) of TDP-43 NTD was acquired over time every 5 s in manual mode with only one run of 90 s, fixed cell position 4.20 and attenuator 10 using 1.369 and 1.1481 as refractive index and viscosity, respectively, measured in this new condition. The dead time was generally 10–15 s. The *D*_H_ of TDP-43 NTD was plotted as a function of time and fitted to a linear equation. From each kinetic trace, the fitted line was used to determine the *D*_H_ value of TDP-43 NTD in the intermediate conformation at 0 s and in the unfolded state at 120 s. These values were then averaged and presented as mean ± SEM.

### 2.8 Thermal Denaturation

The unfolding process of TDP-43 NTD at 62 °C was monitored over time with fluorescence, using a 10 mm path length quartz cell with excitation and emission wavelengths of 280 and 319 nm, respectively, under the same condition described above (5 mM sodium phosphate buffer, 50 mM NaCl, 1 mM DTT, pH 7.4) with a final protein concentration of 0.2 mg/mL (18 μM). The same Agilent Cary Eclipse spectrofluorometer described above was used. The excitation and emission slits were 5 nm. Kinetic traces were fitted to eq 1. Data were also collected from 20 to 42 °C, blank subtracted, plotted versus temperature and fitted to a linear equation to extrapolate the fluorescence value of the native state at 62 °C.

### 2.9 Statistics

All values are presented as mean ± SEM with well-defined *n* values, as indicated in every figure legend for every experiment. Student *t* test was used to assess whether a difference between two values was significant. A *p* value lower than 0.05, 0.01, and 0.001 was considered to be significant (*), highly significant (**) and very highly significant (***), respectively.

## 3 Results

### 3.1 Purified TDP-43 NTD Is Pure and Oligomeric

**Figure 1:**
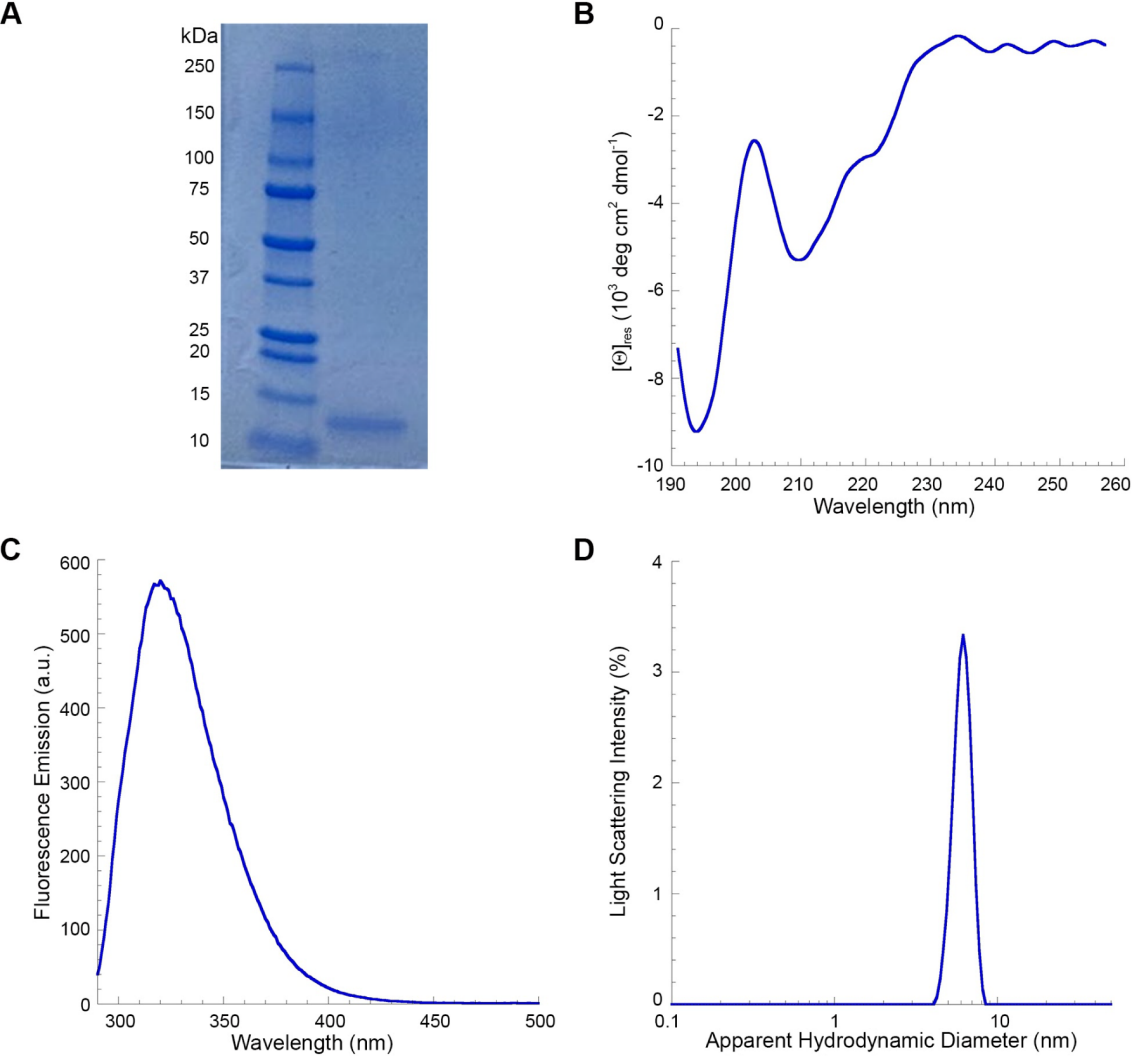
Structural and oligomeric characterization of purified TDP-43 NTD. SDS-PAGE (A), far-UV CD spectra (B), intrinsic fluorescence spectra (C), and DLS distribution (D) of purified TDP-43 NTD at 0.5 mg/mL (45 μM) in 5 mM sodium phosphate buffer, 50 mM NaCl, 1 mM DTT, pH 7.4, 25 °C. The *D*_H_ value is 6.0 ± 0.3 nm (mean ± SEM, *n* = 3).

Purified TDP-43 NTD comprises a 22-residue 6x His-tag followed by TEV protease cleavage site and the first 77 residues of TDP-43, for a total of 99 residues. It was purified following a previously published protocol^35^ with subsequent verification of its purity and proper folding. SDS-PAGE revealed that it is pure, revealing a single band at approximately 11 kDa (Figure 1A), in agreement with the expected value of 11,081.29 Da. Structural characterization of TDP-43 NTD was performed at 0.5 mg/mL (45 μM) in 5 mM sodium phosphate buffer, 50 mM NaCl, 1 mM DTT, pH 7.4, 25 °C. The far-UV CD spectrum exhibited two negative peaks at approximately 195 and 209 nm, along with a distinct small positive band at ca. 233 nm (Figure 1B). The intrinsic fluorescence spectrum featured a single peak at ca. 319 nm (Figure 1C), indicating that TDP-43 NTD is fully folded with the Trp68 residue well buried in the hydrophobic core. Lastly, the light scattering intensity distribution, measured with DLS and plotted as a function of the apparent hydrodynamic diameter (*D*_H_), indicated a monodisperse distribution, with a hydrodynamic diameter of 6.0 ± 0.3 nm (Figure 1D). Expected hydrodynamic diameters for tag-free TDP-43 NTD are 3.23 and 4.28 nm for the folded monomer and dimer, respectively.^34^ Given that our TDP-43 NTD carries an additional unfolded tag, this implies that the native form of TDP-43 NTD exists predominantly as a folded dimer or oligomer. The far-UV CD spectrum, intrinsic fluorescence spectrum and DLS size distribution are in agreement with those previously reported,^34−36^ indicating that our protein domain is correctly folded and natively dimeric or oligomeric.

For one specific experiment, the 22-residue His-tag sequence was enzymatically cleaved, yielding a protein domain of 78 residues, which includes the last serine residue of the tag. Its intrinsic fluorescence spectrum, recorded under identical conditions, demonstrated that it correctly folded, confirming that the additional His-tag does not alter the protein’s conformation. SDS-PAGE analysis of the cleaved TDP-43 NTD confirmed its purity and lower molecular weight, showing a single band at approximately 8 kDa (Figure S1A), consistent with the expected molecular weight of 8615.64 Da.

### 3.2 TDP-43 NTD Unfolding at High Urea Concentration Occurs through the Rapid Formation of an Intermediate State

**Figure 2:**
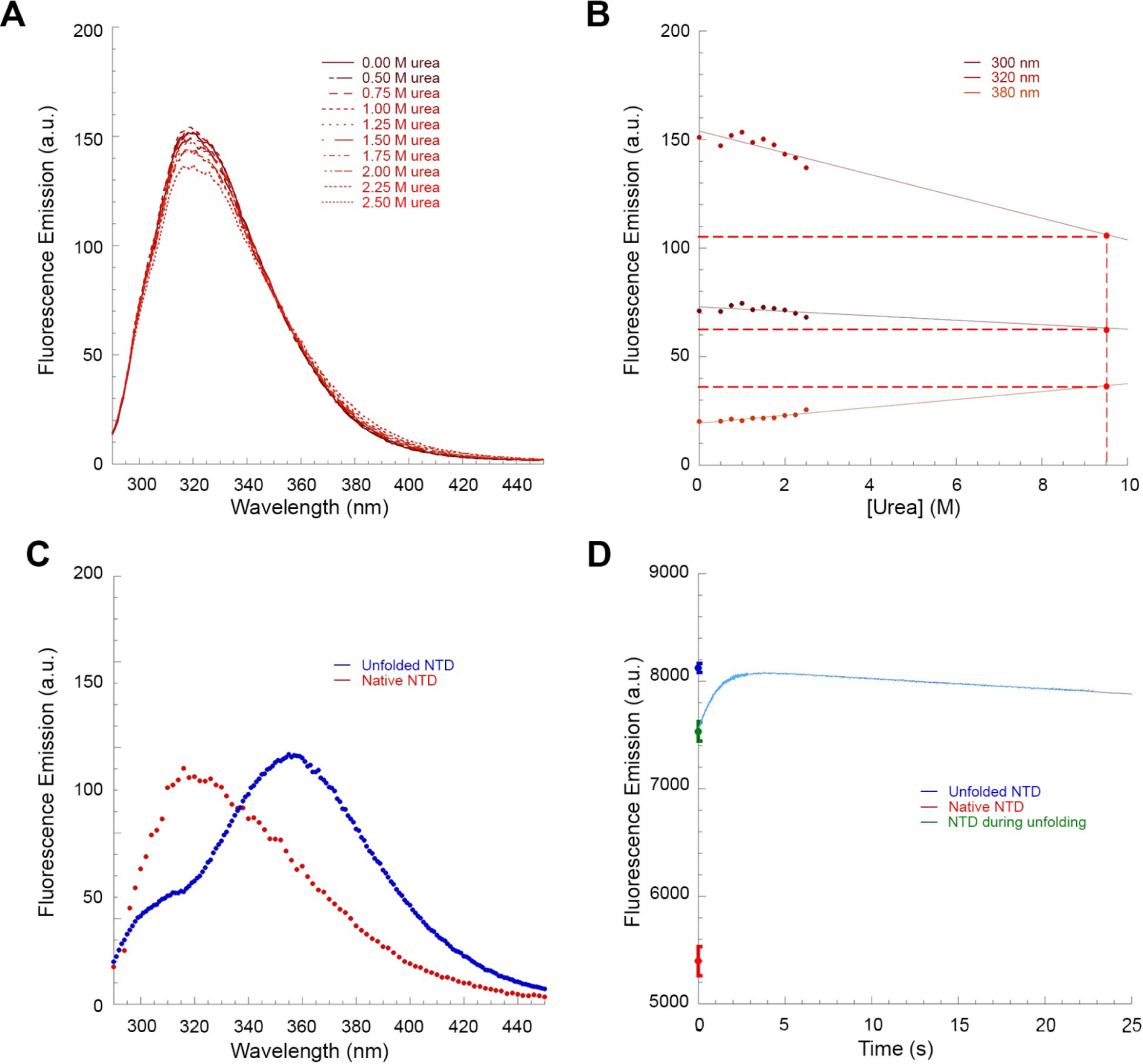
Reconstruction of the fluorescence emission spectra of TDP-43 NTD in 9.5 M urea, and monitoring of its unfolding kinetics in 9.5 M urea using stopped-flow fluorescence spectroscopy. (A) Intrinsic fluorescence spectra of TDP-43 NTD at 0.2 mg/mL (18 μM) with urea concentrations ranging from 0.0 to 2.5 M. (B) Calibration curves (solid red lines) at three representative wavelength values (300, 320, and 380 nm) selected from panel (A). Fluorescence values in 0.0–2.5 M urea were plotted versus urea concentration and fitted with a linear regression to extrapolate the value of the native state in 9.5 M urea (red points and dashed lines). This process was repeated for each wavelength from 290 to 450 nm to reconstruct the intrinsic fluorescence spectrum of native TDP-43 NTD in 9.5 M urea. (C) Fluorescence emission spectra of native TDP-43 NTD in the unfolded state in 9.5 M urea recorded with the spectrofluorometer (blue) and reconstructed spectra of TDP-43 NTD in 9.5 M urea obtained from the extrapolations shown in panel (B) (red). The area under the curve for wavelength >320 nm was then calculated for both spectra and normalized to the fluorescence emission value of TDP-43 NTD in the unfolded state acquired with the stopped-flow device (panel D). (D) Unfolding kinetics (light blue) of TDP-43 NTD in 9.5 M urea monitored using a stopped-flow device. Kinetic traces were blank subtracted. The green point indicates the fluorescence value before the observed exponential phase of unfolding, extrapolated from fitting the kinetic traces to a monoexponential function and averaged (±SEM, *n* = 17) over all the recorded kinetic traces. The red and blue points indicate the normalized fluorescence emission values extrapolated from panel (C) by calculating the area under the spectra (>320 nm) of TDP-43 in 9.5 M urea in the native and unfolded states, respectively (mean ± SEM, *n* = 10 and *n* = 3, respectively).

A common strategy to emphasize the transient formation of unfolding intermediates consists in studying the unfolding process at high denaturant concentrations. Thus, we investigated the denaturation of TDP-43 NTD in 9.5 M urea by means of fluorescence spectroscopy. TDP-43 NTD was initially incubated at 0.2 mg/mL (18 μM) with urea concentrations ranging from 0.0 to 2.5 M, where the protein domain maintains its fold, as assessed by fluorescence and far-UV CD spectra (Figures 2A and S2). Fluorescence spectra were acquired from 290 to 450 nm and the fluorescence emission at each wavelength was subsequently plotted as a function of urea concentration (Figure 2B). The fluorescence values measured at different urea concentrations, but same wavelength, were then fitted to a linear equation, and the resulting straight line (Figure 2B, solid line) was used to extrapolate the fluorescence emission value of native TDP-43 NTD in 9.5 M urea at that wavelength (Figure 2B, dashed line). This analysis was carried out all wavelength values from 290 to 350 nm, thereby yielding the intrinsic fluorescence spectrum of native TDP-43 NTD in 9.5 M urea.

TDP-43 NTD was then diluted into 9.5 M urea to cause its unfolding and its fluorescence spectrum was recorded to enable comparison with the spectrum reconstructed for native TDP-43 NTD in 9.5 M urea (Figure 2C, blue and red spectra, respectively). While the spectrum of TDP-43 NTD in the unfolded state features a fluorescence peak at ca. 356 nm, characteristic of unfolded proteins, the spectrum of the protein domain in its native state displays a fluorescence peak at approximately 320 nm, indicative of a folded protein (Figure 2C). Then, we calculated total fluorescence above 320 nm, which appeared to be 5400 ± 150 au and 8100 ± 50 au for native and unfolded TDP-43 NTD, respectively (Figure 2D, red and blue circles, respectively).

The unfolding reaction of TDP-43 NTD was then monitored with a stopped-flow device, still using tryptophan fluorescence as an optical probe. The use of a rapid mixing device allowed observation of the reaction a few milliseconds after mixing the solutions. The unfolding reaction of TDP-43 NTD was initiated by diluting the folded protein domain to a final protein concentration of 0.03 mg/mL (2.7 μM) and urea concentration of 9.5 M, and the fluorescence emission above 320 nm was recorded (Figure 2D). The unfolding kinetics of TDP-43 NTD show two phases: the first phase is completed in approximately 3 s and features a mean *k*_u_ value of 1.024 ± 0.004 s^–1^, resulting in a rapid exponential increase of fluorescence (Table S1). This phase might correspond to the denaturation of TDP-43 NTD, involving evident conformational changes. The second phase is slower, resulting in a slight linear decrease in fluorescence emission, possibly related to photobleaching, a fluorescence drift or a final relaxation of the protein domain (Figure 2D). The unfolding kinetic trace was normalized so that its value at the end of the first exponential phase corresponded to the area under the curve calculated for unfolded TDP-43 NTD (Figure 2D, light blue) and plotted alongside the values determined for the native and unfolded states (Figure 2D, red and blue circles, respectively). The normalized fluorescence value at 0 s before the major denaturation phase was found to be 7500 ± 100 au (Figure 2D, green circle), which is significantly higher (*p* < 0.0001) than that observed for folded TDP-43 NTD (Figure 2D, red circle).

If TDP-43 NTD were to unfold in a two-state process, the two latter values should be identical, within experimental error. However, since the fluorescence emission value of TDP-43 NTD in the native state differs from that measured during unfolding at 0 s, before the major unfolding phase and after the dead time of the stopped-flow device, it is possible that TDP-43 NTD undergoes a rapid additional conformational change into an intermediate state within the dead time of the stopped-flow device (*ca*. 14 ms) and before the major unfolding phase. This partially unfolded conformation differs in terms of fluorescence emission from both the native and the unfolded states, possibly indicating that it features tryptophan indole moieties not completely solvent exposed but no longer buried in the hydrophobic core.

### 3.3 The TDP-43 NTD Unfolding Intermediate Forms Even at Lower Urea Concentration

**Figure 3:**
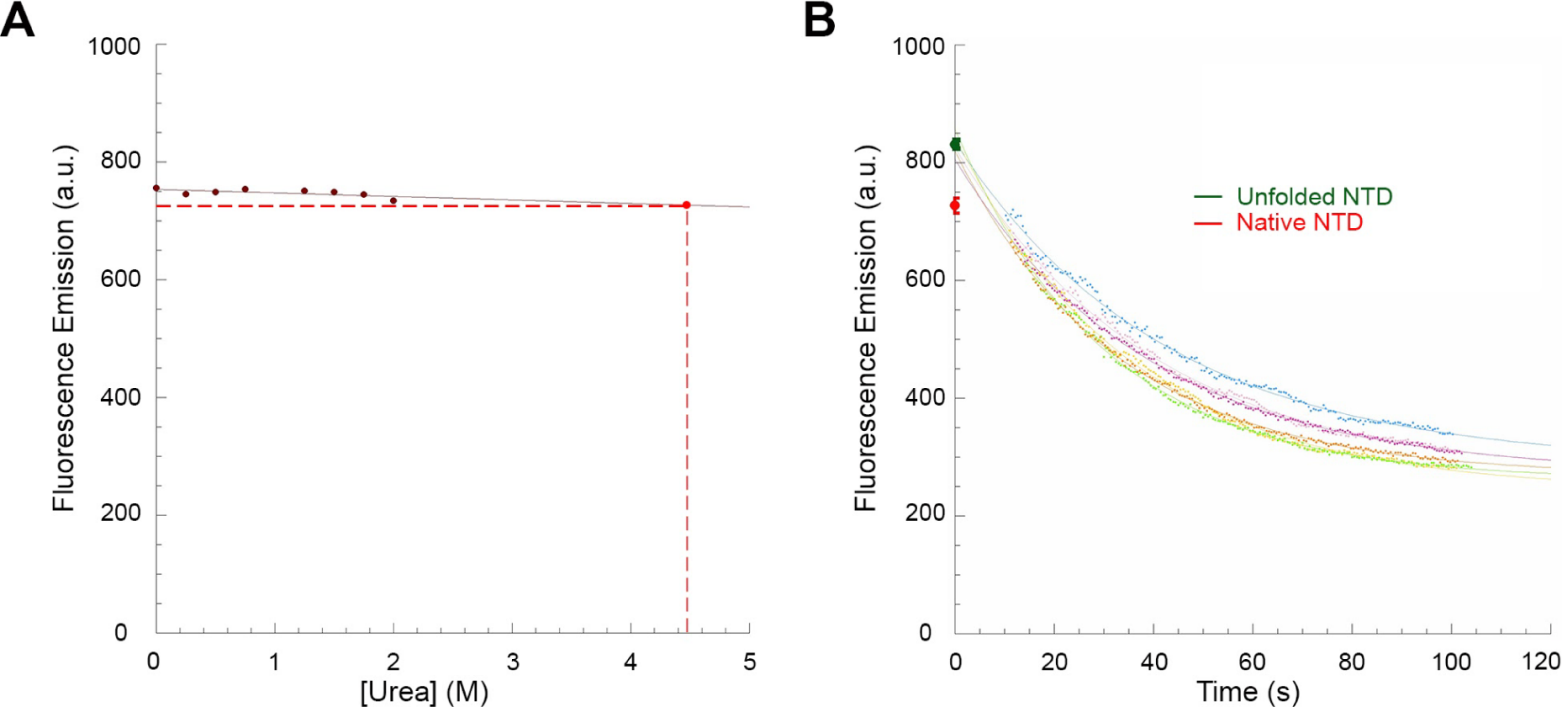
Unfolding of TDP-43 NTD in 4.5 M urea monitored by intrinsic fluorescence spectroscopy. (A) Calibration curve (solid red line) obtained by incubating TDP-43 NTD with urea concentrations ranging from 0.0 to 2.5 M and recording the fluorescence emission at 319 nm. Values were blank subtracted and fitted to a linear equation to determine the extrapolated fluorescence emission value of TDP-43 NTD in the native state in 4.5 M urea (red point and dashed lines). (B) Multiple unfolding kinetic traces of TDP-43 NTD in 4.5 M urea. Kinetic traces were blank subtracted. The green point indicates the fluorescence emission value of TDP-43 NTD before the observed exponential phase of unfolding, extrapolated from fitting the kinetic traces using eq 2 and averaged (±SEM, *n* = 6) over all the recorded kinetic traces. The red point indicates the fluorescence emission value extrapolated from panel (A) (mean ± SEM, *n* = 8).

The unfolding process of TDP-43 NTD was also monitored at a lower urea concentration, namely 4.5 M, in which TDP-43 NTD can still undergo a complete denaturation, but the process is slower. The equilibrium urea denaturation previously acquired indicated an unfolding transition that was substantially complete at 4.5 M urea.^35^ In this regard, TDP-43 NTD was initially incubated at 0.2 mg/mL (18 μM) with urea concentrations ranging from 0.0 to 2.5 M, where the protein domain maintains its fold. Fluorescence emission at 319 nm was recorded for all samples and plotted versus urea concentration (Figure 3A). The data points were then fitted to a linear equation, and the resulting straight line (Figure 3A, solid line) was used to extrapolate the fluorescence emission value of native TDP-43 NTD in 4.5 M urea (Figure 3A, red circle and dashed lines). The unfolding reaction of TDP-43 NTD was then initiated by diluting the protein domain to a final urea concentration of 4.5 M and fluorescence at 319 nm was monitored over time using the same experimental conditions and acquisition parameters used at equilibrium (Figure 3B). Multiple kinetic traces were recorded under the same conditions and visualized (Figure 3B). The observable unfolding of TDP-43 NTD lasts approximately 80 s under these conditions and follows a monoexponential phase, characterized by a marked decay in fluorescence emission with a mean *k*_u_ value of 0.028 ± 0.001 s^–1^ (Figure 3B and Table S1). The fluorescence emission value of TDP-43 NTD at 0 s, that is at the beginning of the observable exponential phase, was then determined using eq 2 for all traces, averaged, and then compared with that of the native state extrapolated from data in the 0.0–2.5 M urea interval (Figure 3B). The fluorescence emission value of the native conformation is 730 ± 15 au, significantly lower (*p* < 0.0001) than that observed before the observed exponential phase of unfolding occurs, which is 830 ± 10 au (Figure 3B).

The experiment was repeated under the same conditions for TDP-43 NTD devoid of the additional MHHHHHHSSGVDLGTENLYFQS sequence fused at the N-terminus (Figures S1B,C). The recorded kinetic traces were very similar to those observed for noncleaved TDP-43 NTD, since they also last approximately 80 s, follow a monoexponential phase, and are characterized by a marked decay in fluorescence emission with a mean *k*_u_ value of 0.030 ± 0.001 s^–1^ (Figure S1C and Table S1), in agreement with the value of 0.028 ± 0.001 s^–1^ observed for the noncleaved protein. The fluorescence emission value of TDP-43 NTD at 0 s was then determined using eq 2 for all traces, averaged, and then compared with that of the native state extrapolated from data in the 0.0–2.5 M urea interval (Figure S1C). The fluorescence emission value of the native conformation is 780 ± 30 au, significantly lower (*p* < 0.001) than that observed before the observed exponential phase of unfolding, which is 940 ± 10 au (Figure S1C).

The difference observed in the intrinsic fluorescence emission value in 4.5 M urea between the native state and the beginning of the observable unfolding process confirms the observation obtained at high urea concentrations and lends support to the presence of an unfolding intermediate forming rapidly within the dead time of the experiment, well before the major phase of unfolding.

### 3.4 The TDP-43 NTD Unfolding Intermediate Is Also Detectable with Far-UV Circular Dichroism

**Figure 4:**
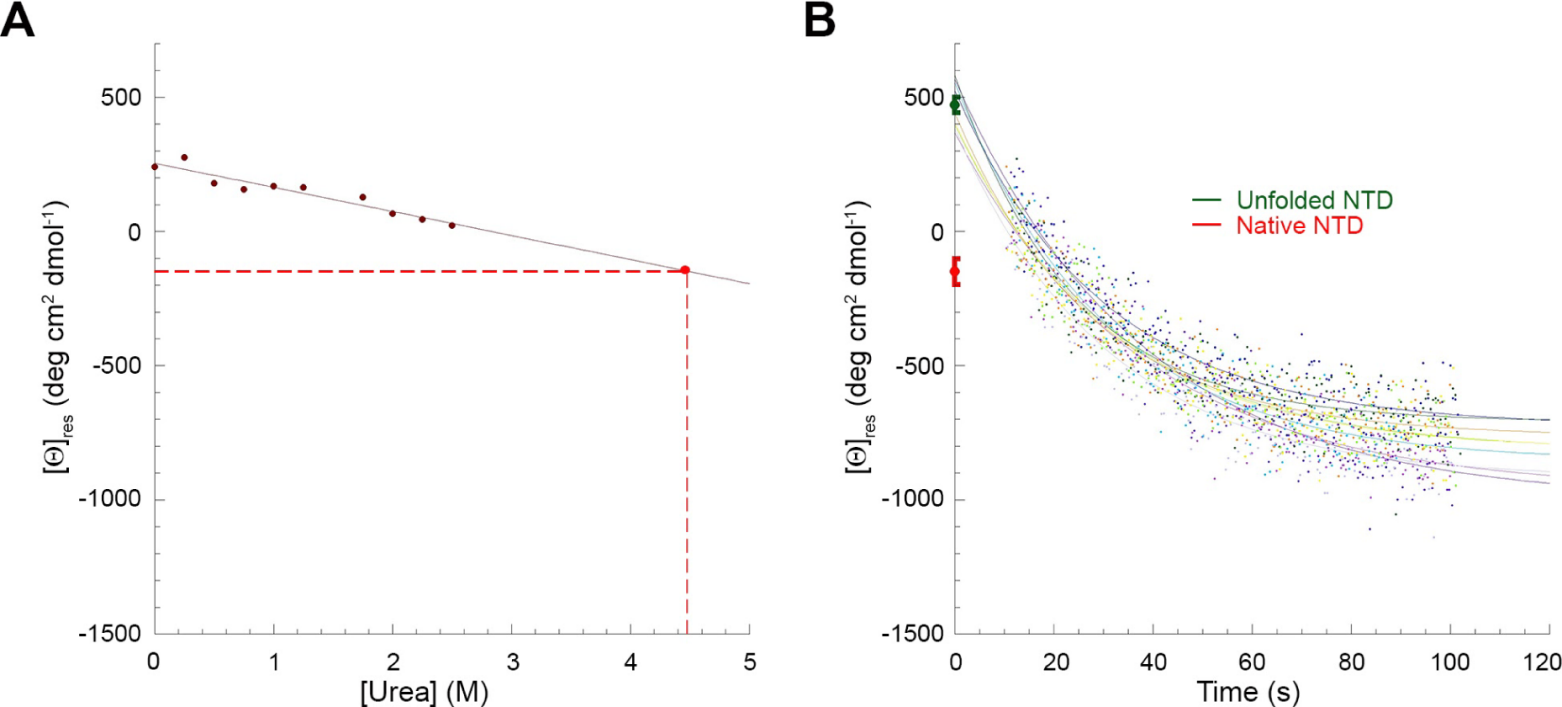
Unfolding of TDP-43 NTD in 4.5 M urea monitored by far-UV CD spectroscopy. (A) Calibration curve (solid red line) obtained by incubating TDP-43 NTD with urea concentrations ranging from 0.0 to 2.5 M and recording the [θ]_res_ at 226–240 nm. Values were blank subtracted, normalized and fitted to a linear equation to determine the extrapolated [θ]_res_ value of TDP-43 NTD in the native state in 4.5 M urea (red point and dashed lines). (B) Multiple unfolding kinetic traces of TDP-43 NTD in 4.5 M urea. Kinetic traces were blank subtracted and normalized. The green point indicates the [θ]_res_ value of TDP-43 NTD before the observed exponential phase of unfolding, extrapolated from fitting the kinetic traces using eq 4 and averaged (±SEM, *n* = 8) over all the recorded kinetic traces. The red point indicates the [θ]_res_ value extrapolated from panel (A) (mean ± SEM, *n* = 10).

The unfolding process of TDP-43 NTD was also monitored using far-UV CD under the same conditions and protein concentration as those used for fluorescence. The mean residue molar ellipticity ([θ]_res_) of TDP-43 NTD, incubated with urea concentrations ranging from 0.0 to 2.5 M, was recorded at 233 nm with bandwidth of 7 nm, averaged and plotted versus urea concentration (Figure 4A). The data points were then fitted to a linear equation, and the resulting straight line (Figure 4A, solid line) was used to extrapolate the [θ]_res_ value of TDP-43 NTD in 4.5 M urea (Figure 4A, dashed line). The unfolding reaction of TDP-43 NTD was initiated by diluting the protein domain to a final urea concentration of 4.5 M under the same conditions and protein concentration. Its [θ]_res_ was monitored over time at 233 nm with bandwidth of ±7 nm, where the distinctive small positive band is located in the spectrum of native TDP-43 NTD without urea, and where there is a notable difference between the spectra of the protein domain in its native and unfolded states (Figure 4B). The observable unfolding of TDP-43 NTD lasts approximately 80 s under these conditions and follows a monoexponential phase, characterized by a significant change in [θ]_res_ with a *k*_u_ value of 0.033 ± 0.002 s^–1^ (Figure 4B and Table S1), in agreement with that measured with fluorescence under the same conditions. Multiple kinetic traces were recorded with far-UV CD and visualized (Figure 4B). The [θ]_res_ value of TDP-43 NTD at 0 s was then determined using eq 4 and compared with that of the native state extrapolated from 0.0 to 2.5 M urea (Figure 4B). The [θ]_res_ value of the native state is −150 ± 50 deg cm^2^ dmol^–1^, significantly lower (*p* < 0.0001) than that observed before the observed exponential phase of unfolding, which is 470 ± 30 deg cm^2^ dmol^–1^ (Figure 4B).

The difference observed in the [θ]_res_ value at 4.5 M urea between the native state and the beginning of the observable unfolding process after the dead time of the measurement, parallels that obtained with intrinsic fluorescence and further confirms the transient formation of an unfolding intermediate formed rapidly well before the major phase of unfolding.

### 3.5 The TDP-43 NTD Unfolding Intermediate Is Detectable with the SYPRO Orange Probe

**Figure 5:**
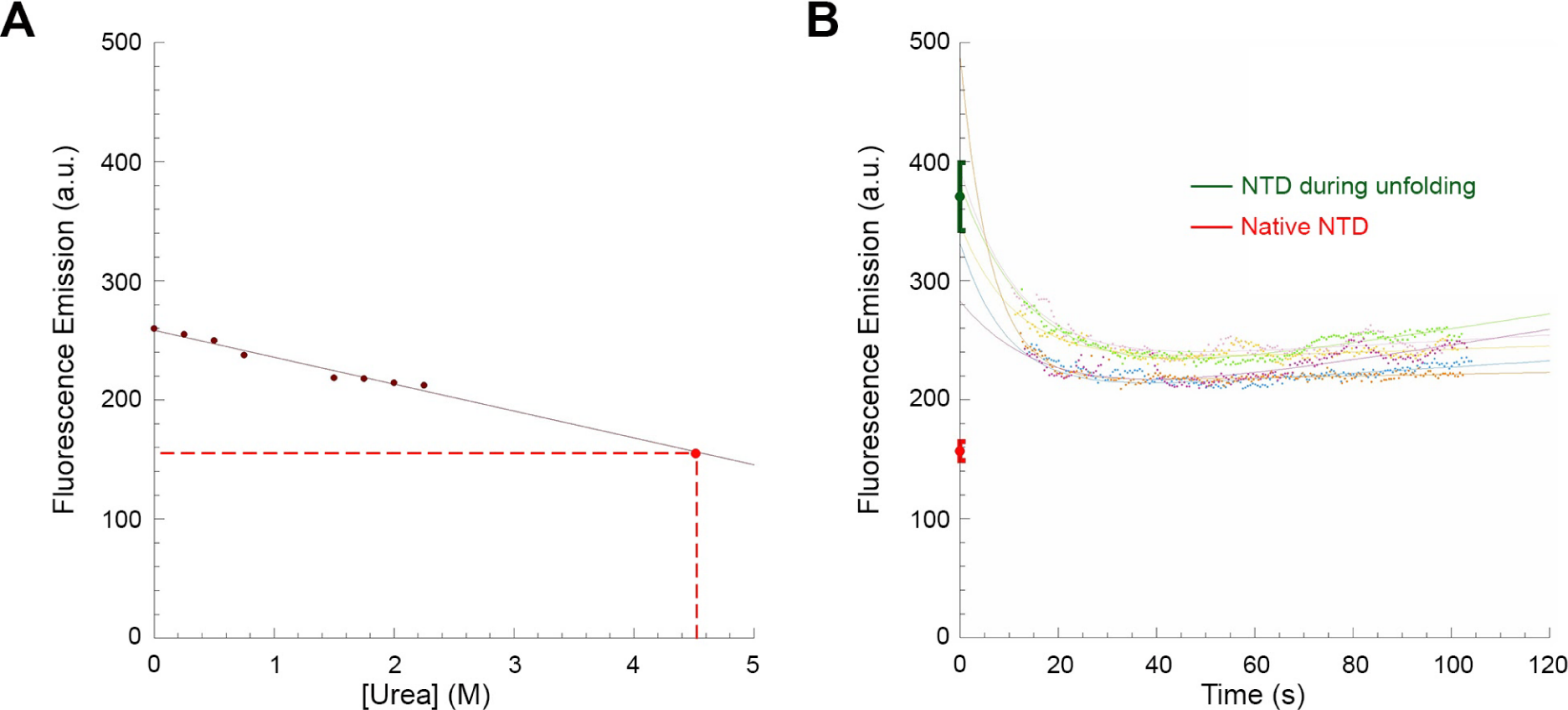
Unfolding of TDP-43 NTD in 4.5 M urea monitored by fluorescence spectroscopy using the SYPRO Orange probe. (A) Calibration curve (solid red line) obtained by treating TDP-43 NTD in the presence of the SYPRO Orange probe with urea concentration ranging from 0.0 to 2.5 M and recording fluorescence signal at 595 nm (excitation of 472 nm). Values were blank subtracted and fitted to a linear equation to determine the extrapolated fluorescence emission value of the SYPRO Orange probe when TDP-43 NTD is in the native state in 4.5 M urea (red point and dashed lines). (B) Unfolding kinetics of TDP-43 NTD in 4.5 M urea in the presence of the SYPRO Orange probe. Kinetic traces were blank subtracted. The green point indicates the initial fluorescence value of SYPRO Orange before TDP-43 NTD undergoes the observed exponential phase of unfolding, extrapolated to 0 s from fitting the kinetic traces using eq 1 and averaged (±SEM, *n* = 6) over all the recorded kinetic traces. The red point indicates the fluorescence emission value extrapolated from panel (A) (mean ± SEM, *n* = 8).

Unfolding of TDP-43 NTD was also monitored in the presence of the SYPRO Orange probe, which increases its fluorescence when it binds to exposed hydrophobic regions, which typically become more accessible during the unfolding process.^39^ TDP-43 NTD was initially incubated with SYPRO Orange under the same conditions and protein concentration used for fluorescence and far-UV CD, with urea concentrations ranging from 0.0 to 2.5 M, where the protein domain maintains its fold. Fluorescence emission of SYPRO Orange at 595 nm (excitation at 472 nm) was plotted against urea concentration. The data points were then fitted to a linear equation (Figure 5A, solid line) to extrapolate the fluorescence value of SYPRO Orange in 4.5 M urea when TDP-43 NTD is in its native form, and found to be 160 ± 10 au (Figure 5A, dashed line).

The unfolding reaction of TDP-43 NTD was initiated in the presence of the fluorescent probe by diluting the protein domain to a final urea concentration of 4.5 M under the same conditions, and the SYPRO Orange fluorescence emission at 595 nm was monitored over time (Figure 5B). The SYPRO Orange fluorescence change during TDP-43 NTD unfolding was found to be more rapid than that observed in the absence of the probe in Section 3.3, and lasts approximately 20 s (Figure 5B). A possible reason for this rapid change may be a mild destabilization caused by the probe itself, which binds to hydrophobic groups and may therefore accelerate unfolding, particularly when an unfolding intermediate forms very rapidly. Control experiments monitoring the intrinsic fluorescence of TDP-43 NTD in the presence of the probe confirmed this acceleration to an extent that made it uncertain to determine its *k*_u_ value. Despite this uncertainty, the obtained kinetic traces were fitted again with eq 1 and the resulting fitted curves were used to extrapolate the fluorescence emission values of SYPRO Orange at 0 s, before TDP-43 NTD undergoes the major phase of unfolding in 4.5 M. The average value was found to be 370 ± 30 au, which is significantly higher (*p* < 0.0001) than that observed for SYPRO Orange when TDP-43 NTD is in its native state, which is 160 ± 10 au (Figure 5B and Table S1). It is also higher than that of the unfolded state at the end of the kinetic traces (Figure 5B). This analysis confirms the rapid formation of an unfolding intermediate with more exposed hydrophobic clusters compared to the native state and even relative to the unfolded state, where hydrophobic groups are more dispersed and bind to the probe less efficiently.

### 3.6 The TDP-43 NTD Unfolding Intermediate Has a Native-like Hydrogen/Deuterium Exchange Pattern

**Figure 6:**
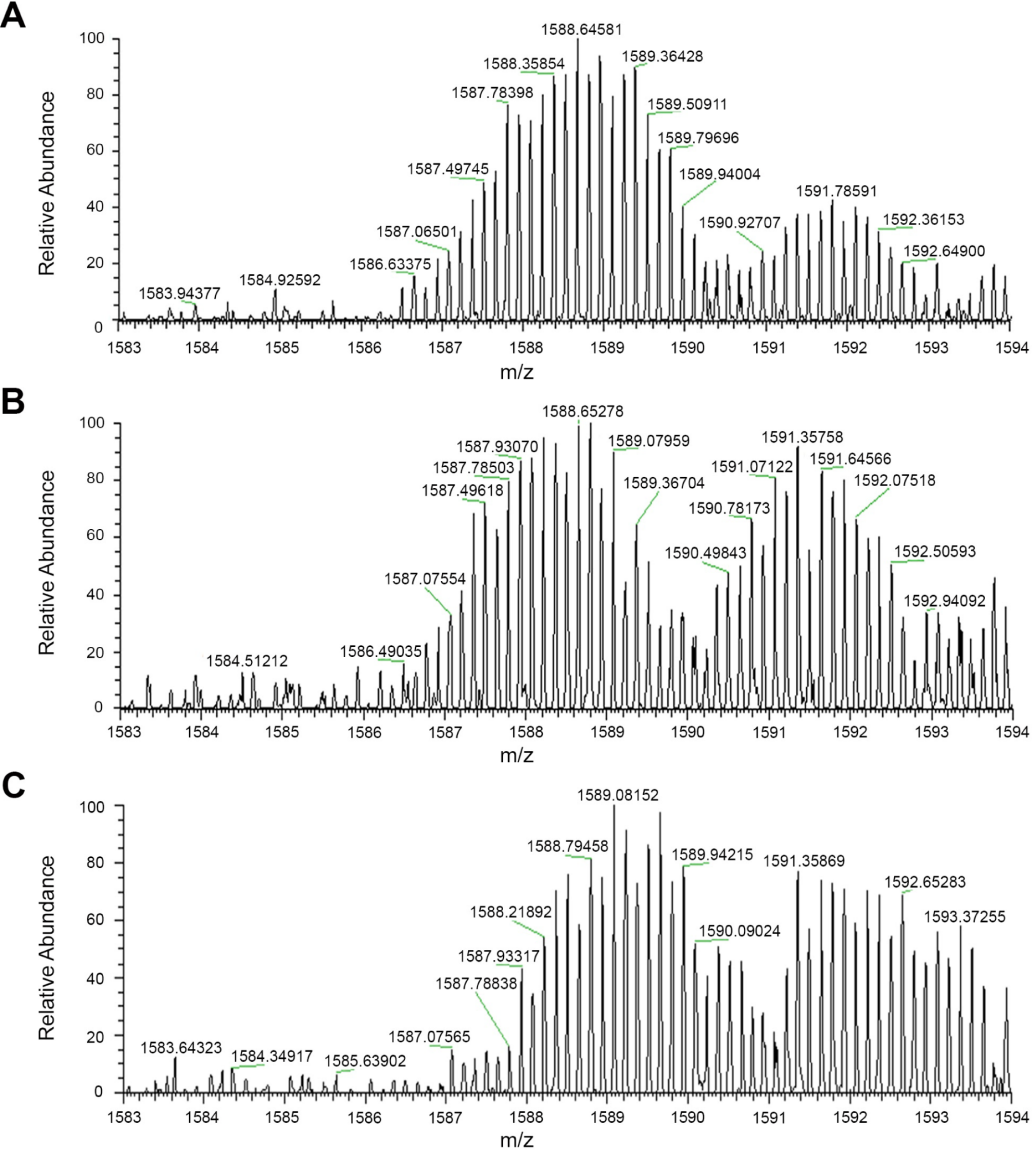
TDP-43 NTD in the native, intermediate, and unfolded state monitored by HDX-MS. Mass spectra of TDP-43 NTD after HDX for 15 s in 1:1 D_2_O (A), 15 s in 1:1 D_2_O and 4.5 M urea-*d*_4_ (B), and 3 min in 1:1 D_2_O and 4.5 M urea-*d*_4_ (C).

To further investigate the structure of the unfolding intermediate, we exploited HDX-MS. First, TDP-43 NTD was diluted at 0 °C in a 1:1 (v/v) with D_2_O, to enable the H-D exchange. After 15 s, the exchange reaction was quenched with 1% TFA and the protein sample was immediately infused into the mass spectrometer. The relative abundance was plotted versus the *m*/*z* ratio (Figure 6A). The mass spectrum of TDP-43 NTD after H-D exchange reveals two major Gaussian distributions with *z* = 7, showing maximum *m*/*z* peaks at approximately 1588.6 and 1591.9, respectively (Figure 6A). Given the presence of NaCl in the sample, the second Gaussian distribution can be attributed to the ionization by sodium ions (sodium adduct), as the two distributions differ by ca. 23 Da [(1591.9–1588.6) × 7], corresponding to the atomic weight of sodium. In each Gaussian distribution at *z* = 7, the individual peaks differ by *m*/*z* values of ca. 0.145, indicating that they represent progressive events of H-D exchange by one H atom.

Subsequently, TDP-43 NTD was diluted at 0 °C in a 1:1 (v/v) ratio with urea-*d*_4_, to a final concentration of 4.5 M. The reaction was quenched with 1% TFA after 15 s, to capture the mass spectrum of the unfolding intermediate. The resulting mass spectrum at *z* = 7 closely resembles that of the native state, with the two Gaussian distributions showing maximum *m*/*z* peaks at approximately 1588.4 and 1591.5, respectively (Figure 6B). Since the extent of deuterium incorporation in the unfolding intermediate of TDP-43 NTD is more similar to that of TDP-43 NTD in the native state, it can be inferred that this intermediate adopts a more native-like conformation.

After 3 min in urea, the mass spectrum of the fully unfolded TDP-43 NTD shows a right shift in the two major Gaussian distributions, with maximum *m*/*z* peaks at approximately 1589.4 and 1592.6, indicating further deuterium incorporation (Figure 6C).

### 3.7 The Unfolding Arm of the Chevron Plot of TDP-43 NTD Is Curved at High Urea Concentrations

**Figure 7:**
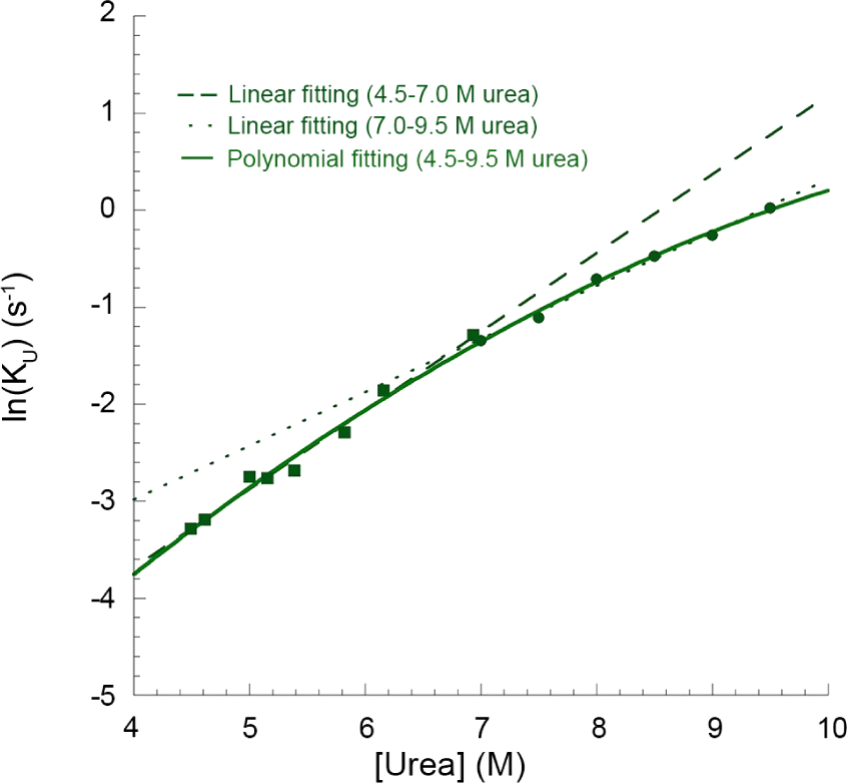
Unfolding arm of the chevron plot of TDP-43 NTD. Natural logarithm of the rate constant (*k*_u_) values of TDP-43 NTD versus urea concentration. Green squares represent ln(*k*_u_) values from 4.5 to 7.0 M urea with their linearly fitted curve (dashed line). These values were taken from a previous study carried out in our laboratory.^35^ Green circles represent ln(*k*_u_) values from 7.0 to 9.5 M urea obtained in this study with their linearly fitted curve (dotted line). All ln(*k*_u_) values from 4.5 to 9.5 M urea are fitted with a polynomial equation of the second order (solid line).

The unfolding kinetics of TDP-43 NTD were subsequently investigated in the presence of increasing urea concentrations ranging from 7.0 to 9.5 M by means of intrinsic fluorescence spectroscopy with a stopped-flow device. Kinetic traces were fitted to eq 1, and the natural logarithm of the obtained rate constant (ln(*k*_u_)) values were plotted versus urea concentration. The ln(*k*_u_) values for urea concentrations ranging from 4.5 to 7.0 M were extracted from a previous study carried out in our laboratory^35^ and reused with those obtained here to generate the unfolding arm of the so-called chevron plot over a wide range of urea concentration, from 4.5 to 9.5 M (Figure 7). As expected, the ln(*k*_u_) values increase upon increasing urea concentration, indicating that as the denaturant concentration increases, TDP-43 NTD unfolds more rapidly (Figure 7). However, the ln(*k*_u_) parameter does not appear to be directly proportional to urea concentration, and the unfolding limb of the chevron plot appears curved rather than linear as the concentration of the denaturant increases from 4.5 to 9.5 M. In particular, the ln(*k*_u_) values exhibit an apparently good linear correlation for urea concentrations between 4.5 and 7.0 M (Figure 7, dashed line), while for concentrations ranging from 7.0 to 9.5 M, they show a correlation with a different linear equation (Figure 7, dotted line). At the same time, when considered altogether, the ln(*k*_u_) values are better fitted to a second order polynomial equation (Figure 7, solid line).

This deviation from linearity occurs under conditions where at least one transient intermediate becomes populated so that the unfolding process does not conform to a two-state model, but rather to a multistate one that involves the accumulation of a partially folded intermediate.^40,41^ Particularly, it is hypothesized that the intermediate rapidly reaches pre-equilibrium with the native state, and the curvature is indicative of an increased stability of the intermediate with increasing denaturant concentration.^41^

### 3.8 The Unfolding Intermediate of TDP-43 NTD Is Observed Even When the Protein Domain Predominantly Exists as a Monomer

The *K*_D_ of the TDP-43 NTD dimer is the ratio between the concentration of the monomer (*M*) raised to the square and that of the dimer (*D*):
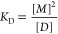
5

The *K*_D_ was found to be 2.4 μM in two independent studies at pH 7.4–7.5.^28,29^ A *K*_D_ of 40 μM was also reported for the oligomer in an isodesmic self-association model at pH 6.8.^27^

**Figure 8:**
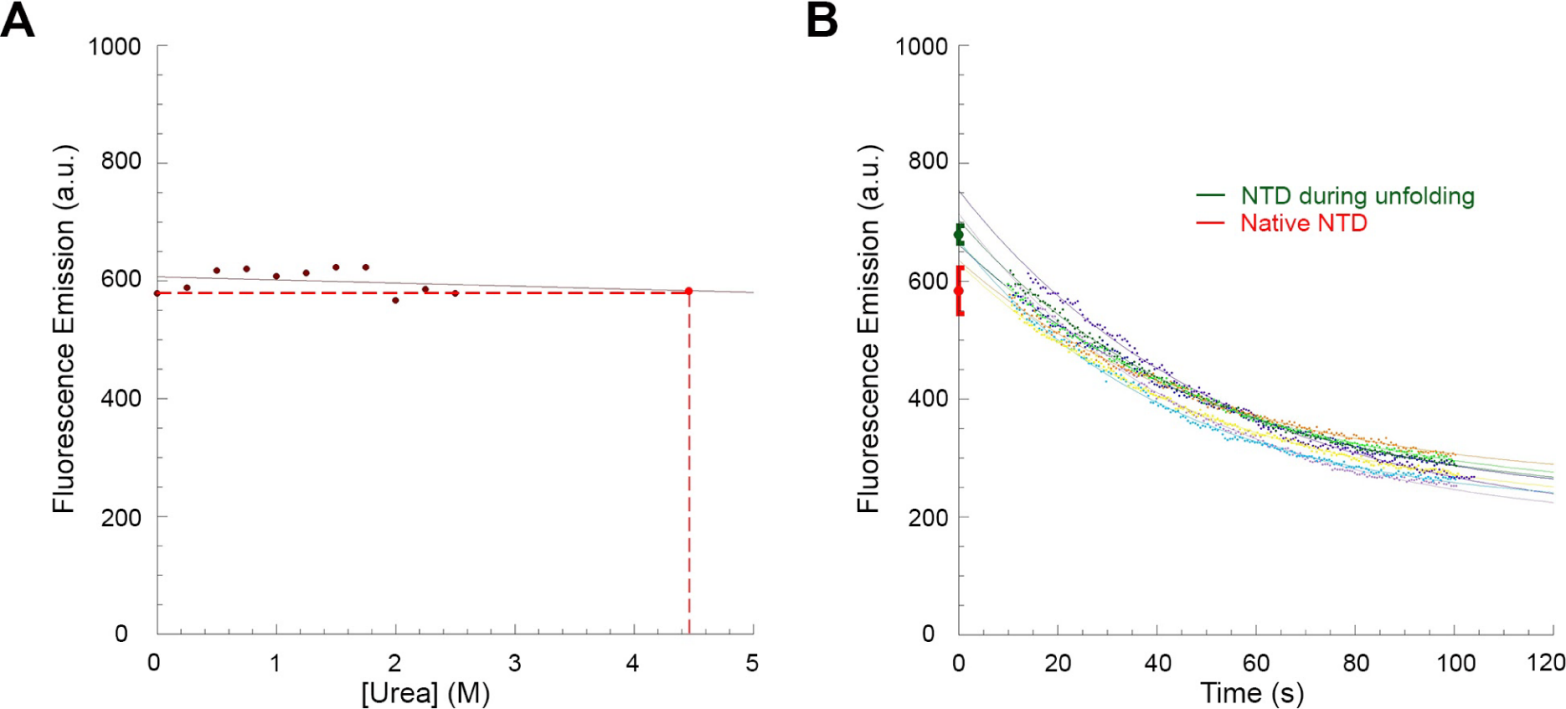
Unfolding of TDP-43 NTD at 0.5 μM in 4.5 M urea monitored by intrinsic fluorescence spectroscopy. (A) Calibration curve (solid red line) obtained by incubating TDP-43 NTD at a concentration of 0.5 μM with urea concentrations ranging from 0.0 to 2.5 M and recording the fluorescence emission at 319 nm. Values were blank subtracted and fitted to a linear equation to determine the extrapolated fluorescence emission value of TDP-43 NTD in the native state in 4.5 M urea (red point and dashed lines). (B) Multiple unfolding kinetic traces of TDP-43 NTD at a concentration of 0.5 μM in 4.5 M urea. Kinetic traces were blank subtracted. The green point indicates the fluorescence emission value of TDP-43 NTD before the observed exponential phase of unfolding, extrapolated from fitting the kinetic traces using eq 2 and averaged (±SEM, *n* = 8) over all the recorded kinetic traces. The red point indicates the fluorescence emission value extrapolated from panel (A) (mean ± SEM, *n* = 11).

In all the experiments presented so far, TDP-43 NTD concentration was 18 μM and the native state present before unfolding was therefore a dimer/oligomer rather than a monomer. To assess whether the appearance of the unfolding intermediate was attributable to the dimer present initially or could be even attributed to the dimer-to-monomer transition, we monitored unfolding at a protein concentration of 0.5 μM, below the different *K*_D_ values, to ensure that TDP-43 NTD is predominantly in the monomer form and, thus, to evaluate whether the formation of the unfolding intermediate of TDP-43 NTD was affected by the initial dimeric state of the protein domain. In particular, the same experiment described in Section 3.3, carried out in 4.5 M urea at 18 μM protein concentration and monitored with intrinsic fluorescence spectroscopy, was repeated at a protein concentration of 0.0055 mg/mL (0.5 μM) (Figure 8A,B). The kinetic traces at lower protein concentration appear slightly slower compared to those recorded at higher protein concentration, with *k*_u_ values of 0.020 ± 0.001 and 0.028 ± 0.001 s^–1^, respectively (Table S1). Nevertheless, when comparing the extrapolated fluorescence emission value of TDP-43 NTD at 0 s in 4.5 M urea and that of the native form extrapolated from urea concentrations ranging 0.0 and 2.5 M urea, a difference is still evident. Indeed, the fluorescence value of TDP-43 NTD in the native conformation is 580 ± 40 au, which is lower (*p* < 0.05) than that observed before unfolding occurs, which is 680 ± 15 au (Figure 8B). These findings indicate that the unfolding intermediate of TDP-43 NTD forms, albeit monomeric, even when the protein domain predominantly exists in the monomer form prior to unfolding and that its formation does not appear to be influenced by the initial oligomerization state of the protein domain.

### 3.9 The Unfolding Intermediate of TDP-43 NTD Is a Partially Folded Dimer

**Figure 9:**
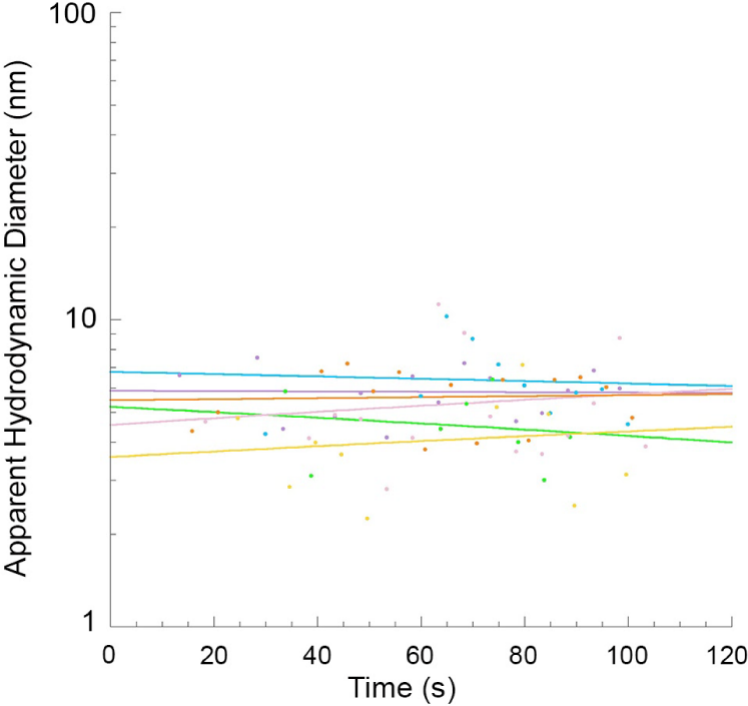
Dimerization/oligomerization state of TDP-43 NTD in 4.5 M urea monitored during unfolding. Time courses of hydrodynamic diameter (*D*_H_) of TDP-43 NTD in 4.5 M urea plotted as a function of time in six independent traces. *D*_H_ values over time from the same experiment were fitted with a linear equation, which was used to extrapolate the *D*_H_ values of TDP-43 NTD before denaturation (at 0 s) and at the end of the recording (at 120 s). Such values were then averaged (±SEM, *n* = 6).

We then aimed at determining the oligomeric state of the TDP-43 NTD during the unfolding process, with a particular focus on the unfolding intermediate. In this regard, dimeric/oligomeric TDP-43 NTD at a concentration of 0.5 mg/mL (45 μM), well above the dimer *K*_D_, was incubated with 4.5 M urea and its *D*_H_ was monitored over time using DLS in six independent traces and with recording every 5 s (Figure 9). This experiment is rarely executed in protein folding and unfolding studies because the rapidity of the reaction makes it impossible to determine in real time the change of *D*_H_, which requires time. The *D*_H_ of TDP-43 NTD remains approximately constant throughout the unfolding process, with values over time fitting satisfactorily with a linear equation. The six recorded kinetic traces did not show a significant decrease or increase with time (Figure 9). The resulting fitting curves were used to determine the *D*_H_ value of TDP-43 NTD before the major phase of unfolding at 0 s, when the protein adopts the unfolding intermediate, and at the end of the recording at 120 s, when the protein domain is an unfolded monomer, as assessed with the other techniques. The obtained *D*_H_ values were 5.2 ± 0.4 nm and 5.3 ± 0.4 nm, respectively, indicating that TDP-43 NTD maintains its *D*_H_ throughout the entire process, as it transitions from the unfolding intermediate to an unfolded monomeric state.

In light of the fact that TDP-43 NTD in the native form exists predominantly as a folded dimer with a *D*_H_ of 4.8 ± 0.3,^35^ which is very similar to that of the unfolded monomeric state estimated theoretically^42^ and measured experimentally,^35^ it can be deduced that TDP-43 NTD maintains a partially folded dimeric structure at the onset of the major phase of unfolding, concurrent with the formation of the unfolding intermediate. Indeed, if one were to hypothesize that the intermediate assumed a compact monomeric form, it would be expected that the *D*_H_ of TDP-43 NTD would increase over time, as the theoretical *D*_H_ of an unfolded monomer is larger than that of a compact monomer. Additionally, the *D*_H_ of the unfolding intermediate of TDP-43 NTD should be lower than that of the native dimer. Since these expectations are not met, it can be concluded that the unfolding intermediate of TDP-43 NTD remains a partially folded dimer, similar to the native state.

### 3.10 Thermal Unfolding of TDP-43 NTD Does Not Lead to the Formation of an Unfolding Intermediate

**Figure 10:**
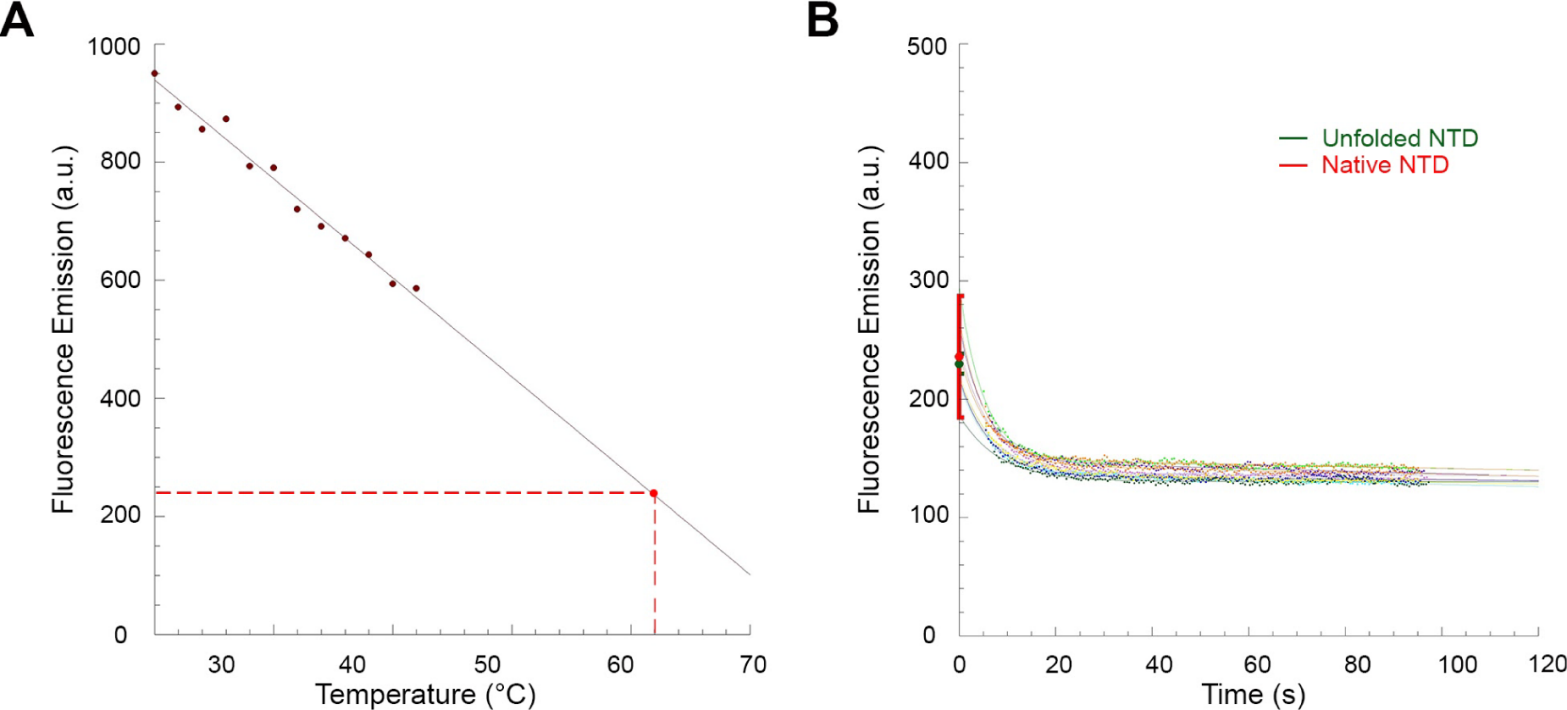
Unfolding of TDP-43 NTD at 62 °C without urea monitored by intrinsic fluorescence spectroscopy. (A) Calibration curve (solid red line) obtained by incubating TDP-43 NTD at temperatures ranging from 20 to 42 °C without urea and recording the fluorescence emission at 319 nm. Values were blank subtracted and fitted to a linear equation to extrapolate the fluorescence emission value of the native state at 62 °C (red point and dashed lines). (B) Multiple unfolding kinetic traces of TDP-43 NTD at 62 °C without urea. Kinetic traces were blank subtracted. The green point indicates the fluorescence emission value of TDP-43 NTD before the observed exponential phase of unfolding, extrapolated from fitting the kinetic traces using eq 1 and averaged (mean ± SEM, *n* = 12) over all the recorded kinetic traces. The red point indicates the fluorescence emission value extrapolated from panel (A) (mean ± SEM, *n* = 10).

We finally investigated whether the formation of the unfolding intermediate also occurs during a thermal denaturation, or if it is unique to urea-induced chemical denaturation. In this regard, TDP-43 NTD was incubated without urea at 0.2 mg/mL (18 μM), at temperatures ranging from 20 to 42 °C, within which the protein domain maintains its fold.^35^ Fluorescence emission at 319 nm was plotted versus urea concentration and the data points were fitted to a linear equation to extrapolate the fluorescence emission value of native TDP-43 NTD at 62 °C, found to be 240 ± 40 au (Figure 10A).

The unfolding reaction of TDP-43 NTD was then initiated at 62 °C in the absence of urea and fluorescence at 319 nm was monitored over time. Multiple kinetic traces were recorded under the same conditions (Figure 10B). The fluorescence change was complete in approximately 15 s and a mean *k*_u_ value of 0.164 ± 0.007 s^–1^ was found (Table S1). The fluorescence value of TDP-43 NTD at 0 s, before the observed exponential phase of unfolding at 62 °C, was then determined using eq 1 and found to be 230 ± 10 au, which was identical, within experimental error, to that of the native state extrapolated at 62 °C (Figure 10B). This observation indicates that the unfolding intermediate does not form when TDP-43 NTD unfolds at high temperatures in the absence of a chemical denaturant, in contrast to the behavior observed during unfolding at 25 °C and moderate to high denaturant concentrations.

## 4 Discussion

### 4.1 The Importance of Partially Folded States of Proteins to Explore Their Self-assembly

The NMR and X-ray structures of TDP-43 NTD have revealed that this domain is responsible for the self-assembly of the full-length protein via head-to-tail interactions,^26,27^ which were later confirmed with FRET studies.^35^ This process enables TDP-43 to form homodimers and higher order oligomers, which are essential for binding to DNA and RNA to exert its functions and prevent aberrant aggregation.^26,27,43^ However, uncontrolled NTD-driven oligomerization can promote aberrant LLPS of the entire protein,^27,30,31^ and formation of irreversible solid inclusions.^24,32^

Given that pathological aggregates of normally globular proteins primarily form when they partially unfold or undergo structural fluctuations into native-like states,^44−48^ understanding the structural plasticity of TDP-43 NTD is crucial within this context. In fact, TDP-43 NTD has repeatedly demonstrated high structural plasticity, as evidenced by NMR reports showing some differences in spectra and monomer–monomer interactions when recorded under various experimental conditions in which the protein domain is folded.^19,21,23,26,27,33,34^ In addition, the folding process of TDP-43 NTD involves the formation of different conformational ensembles that are transiently populated before reaching the final dimeric/oligomeric fully folded state.^35^ With the addition of moderate concentrations of Sulfobetaine 3–10, TDP-43 NTD adopts a monomeric alternative conformation, which reverts to the native dimeric conformation upon its partial removal.^36^ Such conformational heterogeneity was also previously observed in all-atom MD simulations of TDP-43 NTD unfolding, conducted at high temperature and in the presence of DMSO or urea, showing various stable and metastable structural ensembles differing in their free energy and primarily stabilized by non-native hydrophilic interactions.^37,38^

### 4.2 TDP-43 NTD Populates a Partially Folded Dimer during Unfolding

Unfolding intermediates are rarely observed in experimental studies of protein unfolding, which is generally a monoexponential process in which an unfolding intermediate does not form within the deadtime of the experimental measurements. Downward curvatures in the unfolding arms of Chevron plots are observed for some proteins, but they do not generally arise from the accumulation of an unfolding intermediate: they rather originate from a movement of the transition state along the reaction coordinate over a smooth and wide energy barrier (Hammond effect) or to the presence of two or more distinct transition states that overcome each other’s energy depending on denaturant concentration.^49−51^ Here we showed experimentally that an unfolding intermediate accumulates during the unfolding process of TDP-43 NTD. The evidence is experimental rather than simulation-driven and was obtained under conditions of neutral pH, room temperature, and moderate and high urea concentrations, rather than the highly nonphysiological conditions used previously in MD simulations.^37,38^

The investigation of the unfolding of TDP-43 NTD under high urea concentrations by means of intrinsic fluorescence spectroscopy coupled to a rapid mixing instrument has revealed the presence of such intermediate conformation. This ensemble forms rapidly, within 500 ns as detected by MD simulations at high temperatures^37,38^ and was evident after the 14 ms dead time of our stopped-flow device at 25 °C. Notably, the fluorescence emission value observed at 0 s, before the main unfolding phase and after the dead time of the instrument, is significantly lower than that of the native state measured on the spectrofluorometer, extrapolated to the same urea concentration and normalized to the data obtained at the stopped-flow device. If TDP-43 NTD were to unfold following a two-step model, these two values would be identical, within experimental error. This scenario is also present at lower urea concentrations. When monitoring the process with intrinsic fluorescence, but also far-UV CD and SYPRO Orange fluorescence, the intrinsic fluorescence emission, [Θ]_res_ and SYPRO Orange fluorescence values at 0 s were significantly higher than those observed for the native state, further supporting the hypothesis of the presence of an unfolding intermediate. Evidence for the presence of an intermediate in the unfolding process of TDP-43 NTD also comes from the unfolding arm of the chevron plot, where a downward curvature is observed and all ln(*k*_u_) values versus urea concentration are best fitted with a polynomial equation of the second order instead of a single linear equation.

The SYPRO Orange results indicate that the unfolding intermediate, compared to the native state, likely adopts a more open conformation with more exposed hydrophobic residues, explaining the significant differences in fluorescence emission. These experimental results confirm previous findings obtained by MD simulation, showing that the solvent accessible surface area (SASA) of the Trp68 residue side chain and possibly other hydrophobic residues increases after 300 ns of simulations at both 400 and 450 K.^37,38^ The CD results suggest that the β-sheet has a different twist because in the monitored wavelength region the spectrum has a positive peak arising from this structural feature.^35^ This is again in agreement with the results previously obtained with MD simulations showing that after 300 ns the secondary structure has significantly changed and non-native contacts have formed.^37,38^ As the unfolding proceeds toward the fully unfolded state, a significant decrease in [Θ]_res_ is detectable, paralleling the loss of β-strands observed previously in the MD simulations, which unfolded preferentially in urea rather than DMSO.^37,38^ HDX-MS results have revealed that the unfolding intermediate adopts a degree of protection from HDX that is more similar to that of the native state, rather than that of the fully unfolded state. Since the degree of protection depends mainly on the hydrogen bonding of the secondary structure elements and secondarily on the burial from the solvent, this finding further emphasizes that the intermediate state has a compact secondary structure with possible distortions and non-native contacts, suggesting that it retains overall key features of the native fold even as it progresses through the unfolding process.

TDP-43 NTD unfolding monitored by DLS has highlighted that the protein domain maintains a dimeric state in the unfolding intermediate state, before transitioning into the fully unfolded monomeric state. When unfolding was studied at a protein concentration lower than the *K*_D_ reported for dimer dissociation,^27−29^ a significant difference between the fluorescence of TDP-43 NTD extrapolated kinetically at 0 s and that of the native state was still observable, indicating that the intermediate forms, albeit monomeric, even under conditions in which TDP-43 NTD is predominantly monomeric, suggesting that it is not dependent on its oligomeric state.

On the other hand, when the unfolding process of TDP-43 NTD is initiated by high temperatures, rather than moderate or high denaturant concentrations, a difference between the fluorescence of TDP-43 NTD extrapolated kinetically at 0 s and that of the native state was not observed. This indicates that the mechanism of thermal denaturation of TDP-43 NTD differs fundamentally from that of chemical denaturation, with an absence of an unfolding intermediate under thermal denaturation. It also suggests that the energy landscape of TDP-43 NTD is simplified at high temperatures in the absence of chemical denaturants, likely favoring a more direct transition from the native to the unfolded state.

### 4.3 The Unfolding Partially Folded Dimer Is Distinct from Previously Detected Conformational States

One of the peculiarities of the partially folded state observed in this work, is that it can be detected during unfolding kinetics, either spectroscopically (i.e., from fluorescence and CD data) or kinetically (i.e., from the chevron plot). In our previous research, we already detected a set of different transiently populated conformational ensembles of NTD, including a collapsed state (CS) and an on-pathway folding intermediate (I).^35^ Yet, these were identified during refolding. The observation that both the CS and the I found previously form before the major folding energy barrier has been crossed, while the partially folded described here is dimeric and also forming before the major unfolding energy barrier has been crossed, suggests that these three states are distinct. The CS and I previously identified are not native-like, while the present partially folded state presents elements of native-like topology, at least in the two dimerization interfaces.

In principle, in a folding/unfolding equilibrium featuring an obligatory intermediate state (U ⇄ I ⇄ F), one should expect this partially folded conformational ensemble to be populated transiently, and thus detectable, during both folding and unfolding. The folding intermediate I previously identified is not a kinetic trap, but rather a true folding intermediate. However, very rarely partially folded states are detected spectroscopically during unfolding. This apparent discrepancy can be explained when, under conditions promoting unfolding, the free energy barrier that separates the folded (F) and obligatory folding intermediate (I) state is higher than that separating the same intermediate I and the highly stable unfolded (U) state, thus rendering the F → I transition much slower than the I → U step. This prevents significant accumulation, and thus spectroscopic detection, of I during unfolding. Another possible explanation is that parallel pathways are at stake, where the folded protein can unfold following a different route that does not involve formation of an intermediate. It is therefore possible that, under unfolding conditions, the parallel backward pathway not involving conversion into the obligatory intermediate I is quicker.

A second peculiarity of the partially unfolded state populated during unfolding described here, is that it is dimeric/oligomeric. Interestingly, in our previous investigation of NTD refolding, we proposed that, after folding into a folded dimer was complete, the folded dimer was prone to undergo structural rearrangements and populate a native-like dimeric state (F^*^-F^*^). Formation of this F^*^-F^*^ state was suggested by the observation that small concentrations of urea were able to distort the twisting of the β-sheets without inducing full denaturation.^35^ However, the F^*^-F^*^ dimeric state previously detected is distinct from the partially unfolded state populated kinetically during unfolding observed here, because in the former case the [θ]_res_ at 233 nm was found to decrease relative to the native folded dimer and because the Trp68 fluorescence emission did not change, unlike the latter case.

### 4.4 Comparison with Unfolding Intermediates Detected with Other Proteins

A few cases of partially folded conformations transiently detected during unfolding have been reported for other proteins. Horse Apomyoglobin was shown to unfold, in the urea concentration range of 3–4 M, through the transient enhancement of fluorescence. This step causes a rollover in the unfolding limb of the chevron plot and was attributed to the transient formation of a kinetic unfolding native-like intermediate (N’), which subsequently converts into the fully unfolded state.^52^ Similar intermediate conformations were detected during unfolding of sperm whale apomyoglobin.^53,54^ In the case of cytochrome c, a native-like state (N*) forms during unfolding, as evidenced from the detection of an initial deligation step during unfolding, which leads to a folded state with native-like structure but lacking the linkage between iron and Met80.^55^ This N^*^ conformation further converts, prior to full unfolding, into another intermediate state (I*), which appears to be partially unfolded.^55^ A proline-devoid mutant of staphylococcal nuclease (Pro-SNase) forms transiently an intermediate during unfolding, which is different from another intermediate populated during folding and due to a denaturant-induced change in the rate-limiting unfolding step.^56^ Thus, the present identification of a partially folded state populated during unfolding represents a rare but not unprecedented occurrence.

While the identification of a dimeric partially folded conformation transiently populated during unfolding of TDP-43 NTD is novel to our knowledge, a few cases of nonkinetic equilibrium folding/unfolding intermediates have been reported to be dimeric for other proteins and even for TDP-43 NTD, as discussed above.^35^ Bovine serum albumin populates a dimeric conformation at pH 4.2 devoid of disulfide bridges and partially unfolded, as it binds to 1-anilino 8-naphthelene sulfonic acid (ANS) more favorably than at pH 7.0.^57^d-Amino acid oxidase from *Rhodotorula gracilis* is a FAD-binding homodimer. Upon increasing urea concentration, an intermediate molten-globular state forms, which size exclusion chromatography shows to be a multimeric state.^58^ Bovine liver catalase can form enzymatically active and fully folded dimers and tetramers.^59^ However, the enzyme can also form an enzymatically active expanded tetramer and a partially unfolded, enzymatically inactive dimer.^59^

### 4.5 Importance of Structural Plasticity of Proteins for Their Self-assembly

The conformational state identified here for TDP-43 NTD may be relevant and adds further evidence to the high plasticity and ability to populate different conformations observed for this protein domain and possibly promoting its self-assembly. Indeed, several cases of partially folded states populated transiently during folding or unfolding and able to self-assemble have been reported. These include for example the Fyn SH3 domain, which forms an on-pathway intermediate with a disordered C-terminus, with consequent exposure of an aggregation-prone β-strand, an event which triggers self-assembly.^60^ Similar conclusions were drawn for another SH3 domain, from α-spectrin.^61^ β2-microglobulin can populate a native-like folding intermediate with a X-Pro peptide bond in the wrong configuration, which is populated under physiological conditions and highly aggregation-prone.^62−64^ Single molecule experiments carried out on SOD1 illustrate that a set of intermediate states form after the core of the protein has undergone refolding, with some of them branching off the refolding pathway and possibly representing a cross-point between folding and misfolding pathways.^65^ A comparative analysis of a set of ALS-related mutations of profilin-1 showed that the stabilization of a partially folded state induced by pathogenic mutations can enhance the aggregation potential of the protein, possibly contributing to pathogenesis.^66^ The arginine kinase from the sea cucumber *Stichopus japonicus* is a dimeric enzyme that undergoes conformational unfolding and inactivation upon addition of Zn^2+^ salts.^67^ The refolding process follows a biphasic behavior, with formation of an intermediate that can be trapped by Zn^2+^, thus favoring aggregation of the protein through the exposure of hydrophobic clusters.^67^ Finally, the mouse prion protein folds through the accumulation of two intermediates, where the second is native-like, but possesses local disorder and is therefore competent to aggregate.^68^

## 5 Conclusions

The present findings further elucidate the structural plasticity of TDP-43 NTD and provide experimental evidence of the complexity of its unfolding mechanism, which until now had been explored only using all-atom MD simulations under extreme conditions of temperature.^37,38^ The presence of an unfolding intermediate, as well as many folding intermediates previously detected,^35^ along with a clear variability in its monomeric/dimeric/oligomeric state^26−29^ and folded conformation upon subtle variations of the conditions,^36^ suggests that TDP-43 NTD may have folding and misfolding pathways that could be crucial for its function, LLPS and solid aggregation behavior. Understanding these pathways could be important to identify molecular targets for developing therapeutic strategies against TDP-43-related neurodegenerative diseases.

## Data Availability

The data sets used and analyzed during the current study are available from the corresponding author on reasonable request.

## Supporting Information Available

Kinetic parameters, unfolding curves, and spectra for TDP-43 NTD under various experimental conditions (Table S1, Figures S1, and S2) (PDF)

## Abbreviations

ALS: amyotrophic lateral sclerosis
ANS: 1-anilino-8-naphthalenesulfonic acid
CD: circular dichroism
CTD: C-terminal domain
*D*_H_: hydrodynamic diameter
DLS: dynamic light scattering
DMSO: dimethyl sulfoxide
DTT: dithiothreitol
FAD: flavin adenine dinucleotide
FRET: Förster resonance energy transfer
FTLD-TDP: frontotemporal lobar degeneration with tau-negative, ubiquitin-positive inclusions
HT: high tension
LATE-NC: limbic-predominant age-related TDP-43 encephalopathy neuropathological change
LLPS: liquid–liquid phase separation
MD: molecular dynamics
NCI: neuronal cytoplasmic inclusion
NMR: nuclear magnetic resonance
NTD: N-terminal domain
Pro-SNase: proline-devoid mutant of staphylococcal nuclease
RRM: RNA recognition motif
SASA: solvent accessible surface area
SDS-PAGE: sodium dodecyl sulfate–polyacrylamide gel electrophoresis
SEM: standard error of the mean
SOD1: superoxide dismutase-1
TDP-43: TAR DNA-binding protein 43
TEV: tobacco etch virus
TFA: trifluoroacetic acid
