# Supplementary material for: Detection of an Intermediate in the Unfolding Process of the N-Terminal Domain of TDP-43
Source: ACS Omega. 2025 Feb 5;10(6):5616–33. doi: 10.1021/acsomega.4c08617 (PMC11840787; doi:10.1021/acsomega.4c08617)
Supplement: Supplementary file 1 — ao4c08617_si_001.pdf [file ao4c08617_si_001.pdf]

Detection of an intermediate in the unfolding process of the N-terminal domain of TDP-43

Supporting Information

Isabella Marzi [a], Giuseppe Pieraccini[b], Francesco Bemporad [a], Fabrizio Chiti [a,*]

[a] Department of Experimental and Clinical Biomedical Sciences “Mario Serio”, Section of Biochemistry, University of Florence, Viale Morgagni 50, 50134 Florence, Italy
[b] CISM-Mass Spectrometry Centre, University of Florence, Via Ugo Schiff 6, 50019 Sesto Fiorentino, Italy.

* To whom correspondence should be addressed.
E-mail: fabrizio.chiti@unifi.it

Technique and condition	*a*	*b*	A_u	k_u (s^{-1})
Stopped-flow in 9.5 M urea	-9.68±0.06 a.u. s^{-1}	8123±1 a.u.	-591±1 a.u.	1.024±0.004
Intrinsic fluorescence in 4.5 M urea		260±10 a.u.	560±10 a.u.	0.028±0.001
Intrinsic fluorescence of cleaved TDP-43 NTD in 4.5 M urea		410±10 a.u.	490±20 a.u.	0.030±0.001
Circular dichroism in 4.5 M urea		-840±30 deg cm^2 $dmol^{-1}$	1310±40 deg cm^2 $dmol^{-1}$	0.033±0.002
SYPRO Orange fluorescence in 4.5 M urea	0.3±0.1 a.u. s^{-1}	210±10 a.u.	160±30 a.u.	n.d.
Intrinsic fluorescence in 4.5 M urea (0.5 μM NTD)		210±10 a.u.	470±20 a.u.	0.02±0.001
Dynamic light scattering in 4.5 M urea	0.001±0.003 nm s^{-1}	5.2±0.4 nm	n.d.	n.d.
Thermal denaturation in 0 M urea (62 °C)	-0.07±0.01 a.u. s^{-1}	141±2 a.u.	95±8 a.u.	0.164±0.007

Table S1. Kinetic parameters of the unfolding of TDP-43 NTD under various conditions. The table summarizes the kinetic parameters obtained from the unfolding experiments of TDP-43 NTD, measured using various techniques and conditions. The reported parameters include observed linear coefficient (a), observed baseline value (b), observed amplitude of the exponential phase (A_u) and observed rate constant (k_u), as described in equations 1,2,4. Data are presented as mean±SEM.

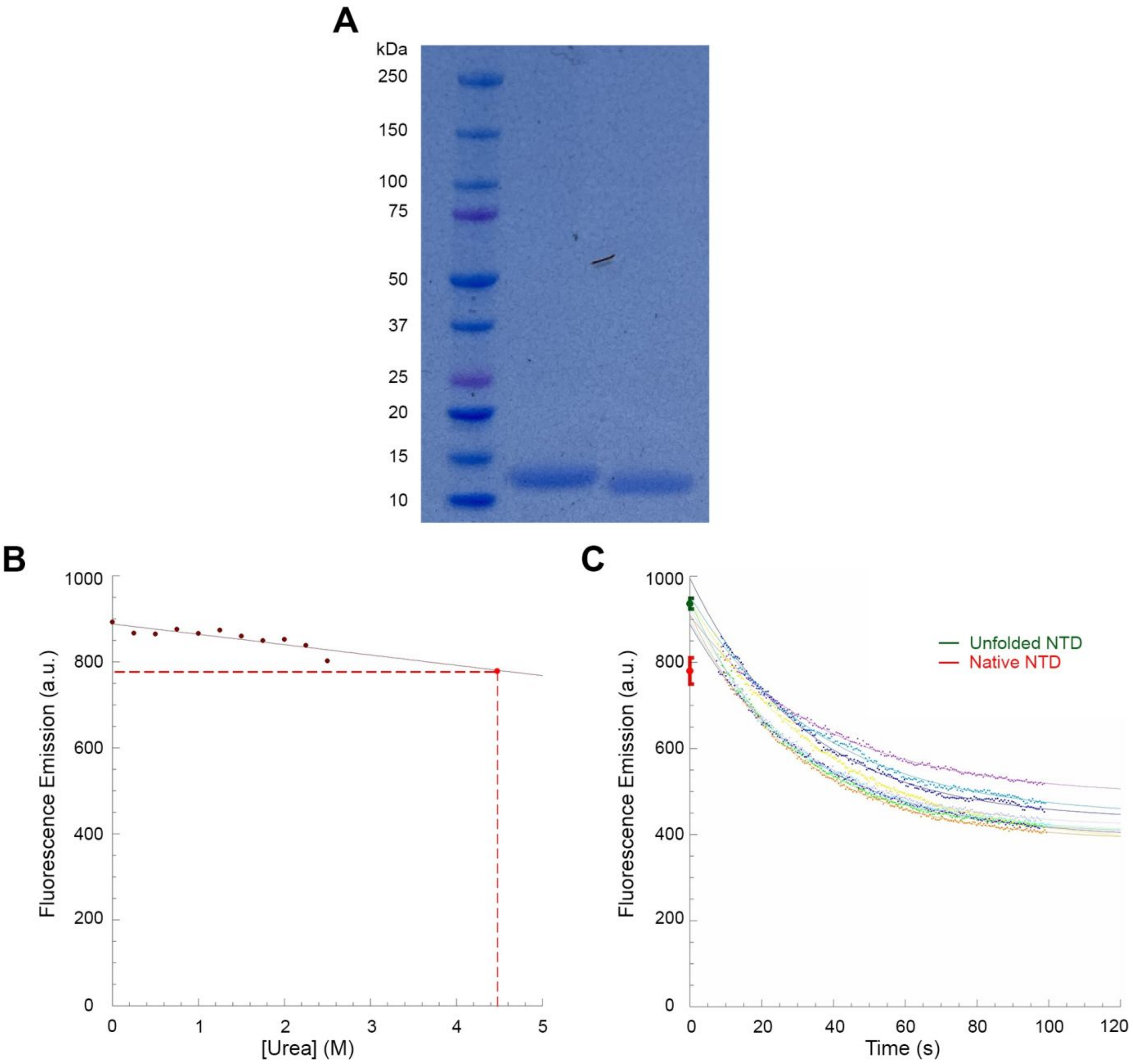

Figure S1. Unfolding of TDP-43 NTD devoid of the His-tag in 4.5 M urea monitored by intrinsic fluorescence spectroscopy. (A) SDS-PAGE of purified TDP-43 NTD before (left) and after (right) removal of the 22-amino acid additional tag. (B) Calibration curve (solid red line) obtained by incubating the cleaved TDP-43 NTD with urea concentrations ranging from 0.0 to 2.5 M and recording the fluorescence emission at 319 nm. Values were blank subtracted and fitted to a linear equation to determine the extrapolated fluorescence emission value of cleaved TDP-43 NTD in the native state in 4.5 M urea (red point and dashed lines). (C) Multiple unfolding kinetic traces of cleaved TDP-43 NTD in 4.5 M urea. Kinetic traces were blank subtracted. The green point indicates the fluorescence emission value of cleaved TDP-43 NTD before the observed exponential phase of unfolding, extrapolated from fitting the kinetic traces using Equation 2 and averaged (±SEM, n=9) over all the recorded kinetic traces. The red point indicates the fluorescence emission value extrapolated from panel A (mean±SEM, n=11).

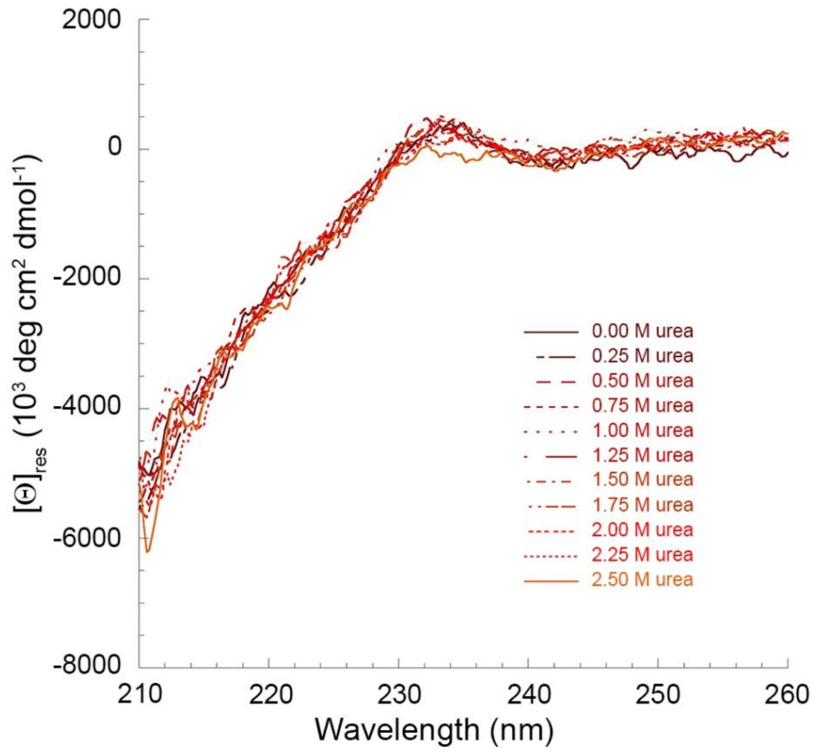

Figure S2. Far-UV CD spectra of TDP-43 NTD in the native state. Far-UV CD spectra of TDP-43 NTD at 0.2 mg/mL (18 μM) with urea concentrations ranging from 0.0 to 2.5 M. Spectra were blank subtracted and normalized to mean residue ellipticity using Equation 3.
